# Alzheimer’s disease: a review on the current trends of the effective diagnosis and therapeutics

**DOI:** 10.3389/fnagi.2024.1429211

**Published:** 2024-08-09

**Authors:** Aimi Syamima Abdul Manap, Reema Almadodi, Shirin Sultana, Maheishinii Grace Sebastian, Kenil Sureshbhai Kavani, Vanessa Elle Lyenouq, Aravind Shankar

**Affiliations:** ^1^Department of Biomedical Science, College of Veterinary Medicine, King Faisal University, Al-Ahsa, Saudi Arabia; ^2^Faculty of Pharmacy and Biomedical Sciences, MAHSA University, Selangor, Malaysia

**Keywords:** Alzheimer’s disease, AD, dementia, diagnosis, treatment, clinical trial

## Abstract

The most prevalent cause of dementia is Alzheimer’s disease. Cognitive decline and accelerating memory loss characterize it. Alzheimer’s disease advances sequentially, starting with preclinical stages, followed by mild cognitive and/or behavioral impairment, and ultimately leading to Alzheimer’s disease dementia. In recent years, healthcare providers have been advised to make an earlier diagnosis of Alzheimer’s, prior to individuals developing Alzheimer’s disease dementia. Regrettably, the identification of early-stage Alzheimer’s disease in clinical settings can be arduous due to the tendency of patients and healthcare providers to disregard symptoms as typical signs of aging. Therefore, accurate and prompt diagnosis of Alzheimer’s disease is essential in order to facilitate the development of disease-modifying and secondary preventive therapies prior to the onset of symptoms. There has been a notable shift in the goal of the diagnosis process, transitioning from merely confirming the presence of symptomatic AD to recognizing the illness in its early, asymptomatic phases. Understanding the evolution of disease-modifying therapies and putting effective diagnostic and therapeutic management into practice requires an understanding of this concept. The outcomes of this study will enhance in-depth knowledge of the current status of Alzheimer’s disease’s diagnosis and treatment, justifying the necessity for the quest for potential novel biomarkers that can contribute to determining the stage of the disease, particularly in its earliest stages. Interestingly, latest clinical trial status on pharmacological agents, the nonpharmacological treatments such as behavior modification, exercise, and cognitive training as well as alternative approach on phytochemicals as neuroprotective agents have been covered in detailed.

## Introduction

**FIGURE 1:**
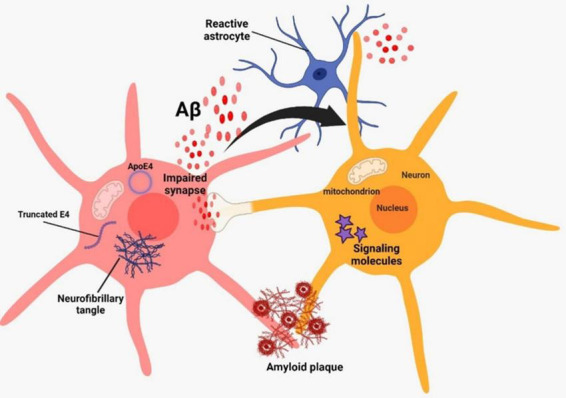
Alzheimer’s pathogenesis based on two classical hallmarks on amyloid beta and neurofibrillary tangles.

Alzheimer’s disease (AD) is a neurodegenerative disorder that leads to the deterioration of brain cells. It is the primary cause of dementia, which is marked by a decline in cognitive abilities and a loss of independence in daily tasks (Porsteinsson et al., 2021). Over 35 million individuals worldwide suffer from AD, and by 2050, the disease’s incidence is predicted to quadruple (Tiwari et al., 2019). Presently, China and the growing Western Pacific, Western Europe, and the United States are the countries or regions most affected by the situation (Li et al., 2022). The World Health Organization (WHO) has designated AD, a condition that mostly affects the elderly and is frequently linked to dementia, as a global health public priority. Because AD progresses in the latent form of the neuropathological process, it presents one of the greatest difficulties to modern neuroscience and medical diagnosis (Nasreddine et al., 2023). The accumulation of abnormal aggregates in the brain called amyloid plaques and tangles of fiber bundles called neurofibrillary (NFTs) are the hallmark of AD (Zhao, 2020). The accumulation of aggregated amyloid beta (Aβ) plaques in the brain begins around 20 years before the onset of cognitive decline in AD, and this can be attributed to either defective clearance of Aβ or excessive production (Serrano-Pozo et al., 2011). The accumulation of hyperphosphorylated tau protein leads to the formation of NFTs, which can be detected a decade to fifteen years before the onset of symptoms (Bateman et al., 2012; Jack et al., 2018; Figure 1).

In 2018, the National Institute on Aging and Alzheimer’s Association (NIA-AA) revised their diagnostic criteria for AD and transitioned from a clinical to a biological perspective on the disease (Jack et al., 2018). AD progresses along a continuum, starting with a phase when there are no symptoms but there is evidence of AD biomarkers (preclinical AD). It then progresses to a stage where there are minor cognitive abnormalities (mild cognitive impairment [MCI]) and/or neurobehavioral alterations (mild behavioral impairment [MBI]), and eventually leads to AD dementia. Several staging approaches have been devised to classify AD along this spectrum (Dubois et al., 2010; Jack et al., 2018). Although the specific definitions of each stage may differ, all of these systems include the assessment of pathological Aβ and NFTs, as well as impairments in cognition, function, and behavior (Dubois et al., 2010; Jack et al., 2018).

**FIGURE 2:**
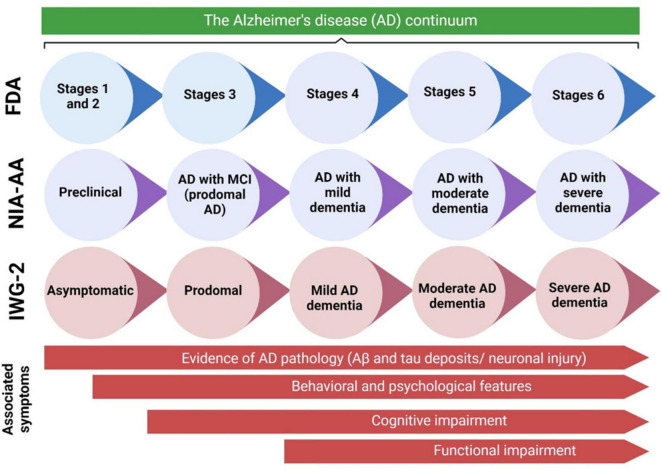
The AD continuum can be categorized into various stages, ranging from preclinical AD to severe AD dementia. The terminology used to describe each stage can vary based on the specific clinical and scientific classifications. This diagram presents an overview of the naming standards employed in the AD community, along with the symptoms associated with each stage of the continuum. Aβ, amyloid beta; AD, Alzheimer’s disease; FDA, Food and Drug Administration; IWG, International Working Group; MCI, mild cognitive impairment; NIA-AA, National Institute on Aging—Alzheimer’s Association. Adaptation and modification from Porsteinsson et al. (2021).

The terminology used to describe each stage may differ across different clinical and research classifications. Figure 2 presents a concise overview of the many naming standards employed in the AD community, along with the corresponding symptoms at each step of the continuum. MBI refers to the development of persistent and significant neuropsychiatric symptoms in individuals aged 50 years or older, before experiencing cognitive decline and dementia (Ismail et al., 2017). Preclinical AD, which is the first stage in the AD progression, involves a prolonged period without symptoms, during which patients show signs of AD pathology but do not experience any cognitive or functional deterioration, and their everyday activities remain unchanged (Dubois et al., 2010; Figure 2). The length of preclinical AD can vary among individuals but generally spans from 6 to 10 years, contingent upon the age at which symptoms first appear (Insel et al., 2019; Vermunt et al., 2019). The likelihood of transitioning from preclinical AD to MCI caused by AD, with or without MBI, is influenced by various characteristics such as age, gender, and apolipoprotein E (ApoE) status (Insel et al., 2019; Vermunt et al., 2019). However, it is important to note that not all persons with underlying AD pathology will eventually acquire MCI or AD dementia (Knopman et al., 2003; Bennett et al., 2006). A recent meta-analysis of six longitudinal cohorts, with an average follow-up period of 3.8 years, revealed that 20% of individuals with preclinical AD developed MCI as a result of AD (Vermunt et al., 2019). In a subsequent investigation conducted by Cho et al. (2021), with an average rate of observation spanning 4 years, it was discovered that 29.1% of individuals diagnosed with preclinical AD experienced a progression to MCI as a result of AD.

In individuals who develop MCI as a result of AD, the first noticeable symptoms usually involve difficulties with short-term memory. This is then followed by a gradual loss in other cognitive abilities in other areas (Kazim and Iqbal, 2016; Figure 2). Individuals with MCI caused by AD may experience difficulties in daily activities such as finding appropriate words (language), remembering recent discussions (episodic memory), completing familiar tasks (executive function), or navigating familiar environments (visuospatial function) (Kazim and Iqbal, 2016; Tolbert et al., 2019). Due to differences in coping techniques and cognitive reserve, patients have diverse experiences and symptoms. Nevertheless, patients generally maintain a reasonable level of independence during this stage, even though they may have minor impairments in function. The outlook for patients with MCI caused by AD can be unpredictable. A study that monitored individuals with MCI caused by AD for an average of 4 years discovered that 43.4% of them developed AD dementia (Cho et al., 2021). Additional research findings indicate that 32.7% and 70.0% of persons diagnosed with MCI caused by AD develop AD dementia within 3.2 and 3.6 years of observation, respectively (Roberts et al., 2018; Ye et al., 2018). Individuals who advance to AD dementia will experience significant cognitive impairments that hinder their ability to engage in social interactions and necessitate help with everyday tasks (Jack et al., 2018). As the condition advances, more pronounced behavioral symptoms will arise, imposing a substantial load on both patients and their caretakers. Ultimately, the disease leads to a profound decline in independence and necessitates constant care.

Early diagnosis of AD is essential in order to facilitate the development of disease-modifying and secondary preventive therapies prior to the onset of symptoms (Nasreddine et al., 2023). There has been a notable shift in the goal of the diagnosis process, transitioning from merely confirming the presence of symptomatic AD to recognizing the illness in its early, asymptomatic phases. Validating biomarkers as accurate indicators of AD pathology would allow them to be utilized as diagnostic tools, eliminating the need for brain samples or autopsies to confirm an accurate diagnosis (Lee et al., 2019). The NIA-AA has classified diagnostic biomarkers for AD into three categories: Aβ-Aβ deposits (A), hyperphosphorylated tau aggregates (T), and neurodegeneration or neuronal damage (N). The ATN categorization based on NIA-AA research framework is displayed in Table 1 (Jack et al., 2018). The AD continuum is associated with one of the following biomarker profiles: A + T- N-, A + T+ N-, A + T+N +, or A + T-N +, regardless of any clinical symptoms (Jack et al., 2018). Evaluation of the ATN profile is conducted using biofluids, such as cerebrospinal fluid (CSF), or imaging techniques, such as Positron Emission Tomography (PET). The objective of the present study is to provide extensive reviews on comprehensive diagnostic and therapeutic approaches grounded in a precisely defined ATN model that corresponds to the AD continuum. Additionally, the details on the most recent clinical trials involving pharmacological agents employed in therapeutic strategies have been presented. In the future, it will be crucial to investigate novel biomarkers that extend beyond the amyloid and tau pathologies, as well as the longitudinal evolution of these biomarkers throughout the course of AD.

**TABLE 1:** Biomarker profiles and categories.

ATN profiles	Category of the biomarker	
A-T-N-	Normal AD biomarkers	
A+T-N-	Alzheimer’s pathologic change	Alzheimer’s continuum
A+T+N-	Alzheimer’s disease	
A+T+N+	Alzheimer’s disease	
A+T-N+	Alzheimer’s and concomitant suspected non-Alzheimer’s pathologic change	
A-T+N-	Non-AD pathologic change	
A-T-N+	Non-AD pathologic change	
A-T+N+	Non-AD pathologic change	

AD, Alzheimer’s disease.

## Diagnostic process

The process of diagnosing AD can be categorized into the subsequent stages: identification, evaluation/differentiation, diagnosis, and treatment. Clinicians must employ suitable diagnostic techniques when examining a patient who is suspected of having AD in its initial phases.

## Identification

In the context of dementia, the initial step of diagnosis does not involve executing tests but rather involves developing a suspicion that a dementia syndrome may be developing (referred to as the trigger phase). An issue that arises with dementia is the hesitancy of certain patients, families, and primary care physicians to make a diagnosis. Dementia is a severe and mostly unchangeable disease that is associated with a significant amount of social disgrace. Physicians may inadvertently hesitate to diagnose a patient with a specific condition (Downs and Bowers, 2008), and family members may gradually assume the social responsibilities of the patient without being fully aware of their actions. This unintentionally shields the patient from worsening in their daily life, but also delays the conscious acknowledgment of the disorder by compensating for the impairments (De Lepeleire et al., 1998; Iliffe et al., 2009). To confirm the presence of symptoms related to AD, the healthcare provider must perform an initial examination on patients who display even minor symptoms. This assessment should utilize a validated tool for detecting early-stage AD as discussed below.

## Evaluation of a memory complaint

### Clinical assessment tool

Clinical assessment tools for evaluating memory complaints often include a combination of interviews, questionnaires, and cognitive tests. These tools help healthcare professionals gather information about a person’s memory concerns and assess their cognitive function. Nasreddine et al. (2023) reported a number of common and recent scales used in early diagnosis of AD include Mini-Cog, (MMSE) Mini-Mental State Examination, and MoCA. Those generally used in primary care and they are varying in sensitivity (Nasreddine et al., 2023). MMSE is used primarily for assessing overall cognitive function including memory-related questions. It assesses orientation, attention, calculation, recall, and language, providing insights into memory and other cognitive domains. Therefore, it is low sensitivity compared to MoCA high sensitivity which assesses various cognitive domains, including memory. It includes tasks related to immediate and delayed recall, as well as other memory-related exercises (Nasreddine et al., 2023). The summarization on the screening tools utilized in the early diagnosis of AD is demonstrated in Table 2.

**TABLE 2:** Summarisation of the screening tools utilized in the early diagnosis of AD.

Screening tools	Cognitive scale				Advantages and disadvantages	References
	Level	Duration	Assessment	Outcome/scoring		
Mini-Cog	The shortest cognitive assessments, consisting of two parts: a three-item recall task and a clock-drawing task.	2–3 min	Primarily assesses two cognitive domains: immediate and delayed recall and visuospatial/executive function (as demonstrated by the clock-drawing task)	It is scored out of 5 points, with 2 points for correct recall and 3 points for a correctly drawn clock. A lower score is indicative of cognitive impairment	Quick and easy to administer, making it a useful tool for initial screening in busy clinical settings. however, it has low sensitivity	Nasreddine et al., 2023
MMSE	Moderately brief cognitive assessment.	5–10 min	It assesses various cognitive domains, including orientation, registration, attention, calculation, recall, language, and visuospatial skills	Scored out of 30 points, with higher scores indicating better cognitive function. A lower score suggests cognitive impairment	Can help differentiate between different types and stages of cognitive impairment. However, it has low sensitivity	
MoCA	Moderately comprehensive assessment	10 min	It assesses multiple cognitive domains, including attention and concentration, executive functions, memory, language, visuospatial skills, abstract thinking, and orientation	Scored out of 30 points, with higher scores indicating better cognitive function. Lower scores are suggestive of cognitive impairment	The MoCA is more sensitive to mild cognitive deficits than the MMSE and provides a broader assessment of cognitive function. It is particularly useful for identifying early-stage AD and MCI	
Mini-Cog	Consist of clock drawing and see CDT	7–8 min	Basically, aimed to detect dementia, besides that repetition of 3 words has no connection	Maximum score 30	Simple and consists of immediate recall words, however, has low sensitivity	Riello et al., 2021
MMSE	The main purpose of this test is to detect dementia in moderate-to-severe stages	3–4 min	Orientation in time and space, perception of speech, and working memory	Maximum score 5	Effective consists of calculation, working memory, and attention, however, has low sensitivity	
MoCA	Specific to MCI	10 min	It is mostly used to detect MCI (especially for those with a MMSE score above 24)	Maximum Score 30	It is sensitive and associated with MMSE	
Mini-Cog	Can be used by primary care and easily preformed with short time	3 min	Superior to MMSE in terms of sensitivity, specificity, positive predictive value, and negative predictive value in detecting MCI	Scored out of 9 points	It is accepted by patients and doctors. Also, higher in sensitivity and specificity to screen patients with dementia	Henneges et al., 2016; Li et al., 2018
MMSE	In clinical practice and research, the most widely used tool for assessing cognitive function	5–10 min	Can provide useful information about AD monitoring and progression, can provide powerful information	A score 25 ≥ normal Score ≤ 26 possible cognitive impairment.	The score correlates with disease progression; however, it is hard for doctors and patients to comprehend what lowering score in MMSE means regarding impairment	
MoCA	Used for screening patients with MCI	10 15 min	It involves attention, executive function, memory, language, visuospatial skills, abstract thinking, calculation, and orientation as cognitive areas.	Scored out of 30	The most frequently employed cognitive function screening scales. High sensitivity to MCI. However, it is not appropriate to be used by health professionals as an outpatient.	

Furthermore, a variety of neuropsychological tests have been used in previous research to assess AD. Recently, the Rey Auditory Verbal Learning Test (RAVLT) is a widely used neuropsychological assessment tool designed to evaluate various aspects of verbal memory and learning in AD and other forms of dementia (Warren et al., 2023). Based on previous investigation studies, the RAVLT performance in individuals with memory complaints reflects well the underlying pathology caused by AD. As a result, RAVLT is an effective early marker for detecting AD in people who have memory problems (Moradi et al., 2017). AD is commonly diagnosed with the clinical dementia rating (CDR) scale. Recently, interventional trials have emphasized the sum of boxes of the clinical dementia rating-sum of boxes (CDR-SB) to track the progression of cognitive impairment (CI) in the early stages of AD. As the stages of predementia progress, researchers have developed practical tools for measuring the deterioration of cognition or daily function. A recent study was conducted in Taiwan by Tzeng et al. (2022) have assessed the predictive value of the CDR-SB and CDR to widely employed AD staging tools, by investigating how they contribute to the process or reversion in individuals without dementia (Tzeng et al., 2022). There were 1,827 participants observed, and during evaluation, a trained physician scored six cognitive or functional domains, including memory, orientation, judgment, community affairs, home hobbies, and personal care, following interviews with both participants and informants. Consequently, performance and function-based information are simultaneously acquired. This study’s significant results deserve special attention. Findings were found such as the CDR-SB tool has an excellent predictive value in detecting the onset of dementia in people without dementia. In addition, an increase in CDR-SB scores was associated with higher conversion rates, and the prediction power of CDR-SB levels was very good. CDR-SB is a reliable and global diagnostic tool. Additionally, it is very sensitive in detecting the disease progression among participants with different levels of disease severity (Tzeng et al., 2022). However, in clinical trials, it is not capable of consistently detecting treatment effects (Wessels et al., 2022).

Other cognitive scale used in clinical practice neuropsychological tests to detect AD is AD Assessment Scale–Cognitive (ADAS-Cog), which measures cognitive deficits, such as memory, language, and praxis. Cogo-Moreira et al. (2023) stated that the ADAS-Cog has been a prominent assessment tool and has been widely used in investigations of AD since its establishment in 1984 (Cogo-Moreira et al., 2023). Due to its wide application, many studies have been conducted to evaluate, improve, and optimize ADAS-Cog for various uses (i.e., ADAS-11 and ADAS-13), as well as to serve as indicator of AD progression (ADAS-Cog) (Kueper et al., 2018; Cogo-Moreira et al., 2023; Warren et al., 2023). Based on a study relating to ADAS-Cog, they hypothesized that different stages can be predicted of AD continuum with ADAS-13. This hypothesis was proven based on a research study that ADAS-11 could effectively distinguish between those with cognitive impairment and those with early AD (Zainal et al., 2016). Similarly, some modifications have been made to ADAS-Cog-11, including assessments of executive function, improved scoring methodology, delayed recall and/or everyday functioning in order to detect early signs of cognitive decline preceding dementia (Kueper et al., 2018). From all these findings shown, employing the ADAS-13 in clinical practice should be used to assess cognitive function when patients present with minor memory problems, as it distinguishes between the levels of cognitive function associated with different stages of AD. Basically, the scores can range from 0 to 70 for ADAS-Cog-11, with higher scores indicating greater cognitive impairment and scores are from 0 to 85 for ADAS-Cog-13, which takes approximately 30–45 min (Clarke et al., 2022).

A comprehensive study done by Warren et al. (2023) which evaluating the ability of the most commonly used neuropsychological tests to screen AD. Moreover, it focuses on its ability to differentiate and distinguish of AD disease (Warren et al., 2023) such as cognitively normal (CN), Subjective Memory Complaints (SMC), and MCI. Basically, the study included a total of 595 participants with AD. The screening tools include The Everyday Cognition Questionnaire (ECog), the Rey Auditory Verbal Learning Test (RAVLT), the Functional Abilities Questionnaire (FAQ), the AD Assessment Scale–Cognitive Subscale (ADAS-Cog), the Montreal Cognitive Assessment scale (MoCA), and the Trail Making test (TMT-B) as summarized in Table 3. Interestingly, the study’s outcomes and results point out that screening tools such as ADAS-13, RAVLT (learning), FAQ, ECog, and MoCA all predicated the progression of AD. Furthermore, TMT-B and the RAVLT were not specific for predicting AD in contrast with ECog that showed a very strong predictor tool into screen AD progression. Finally, the author recommends and suggests using ECog (both versions), RAVLT (learning), ADAS-13, and the MoCA to screen AD in all stages.

**TABLE 3:** Summarisation of advantages and disadvantages of screening tools employed in AD.

Screening tools	Cognitive scale		Reference
	Assessment	Advantages and disadvantages	
The Everyday Cognition Questionnaire (ECog)	Primary tool used to assess everyday cognitive function; it is very sensitive in detecting early stage of AD and its progression. It is a self-report questionnaire that is often administered to individuals, typically with the help of a caregiver or family member who can provide additional insights into the individual’s cognitive abilities	It is very specific to Everyday Memory, Everyday Language, Everyday Visuospatial abilities, and three everyday executive domains including Everyday Planning, Everyday Organization, and Everyday Divided Attention Moreover. ECog designed as questionnaire. ECog has good reliability as well as concurrent, it is sensitive to very early functional difficulties, and is associated with other disease markers such as the presence of amyloid and tau	Farias et al., 2021
The Rey Auditory Verbal Learning Test (RAVLT)	A widely used neuropsychological assessment tool designed to evaluate various cognitive functions, including memory, learning, and recall	RAVLT can be used to detect AD in its early stages. Also, RAVLT is also important in distinguishing AD from psychiatric disorders. widely used in dementia and pre-dementia assessment. Sometimes RAVLT not being able to address temporality.	Parra Bautista et al., 2023
The Functional Abilities Questionnaire (FAQ)	The FAQ consists of a series of questions or items that pertain to various everyday activities. Caregivers are asked to rate the individual’s current level of functioning in these activities, considering any cognitive impairments they may have observed. The items typically cover a range of functional areas, including shopping, finance, communication, transportation.	FAQ has the ability to predict differences in IADL across the AD continuum in early-stage AD, FAQ can distinguish between CN and SMC, and develop scales that emphasize only complex activities of daily living	Warren et al., 2023
Trail Making test (TMT-B)	The TMT-B measures several cognitive functions, the task requires the creation of an ascending pattern of alternating numbers and letters as quickly and accurately as possible. The final score is based on the time taken to complete the task, and participants are advised to correct mistakes as soon as possible.	TMT-B would struggle to accurately categorize individuals with SMC. More specifically, previous studies have indicated that the TMT-B does not have a significant ability to distinguish between individuals who are CN and those with MCI.	Papp et al., 2014; Warren et al., 2023
The Everyday Cognition Questionnaire (ECog)	The ECog can provide valuable insights into an individual’s perceived cognitive difficulties. can help healthcare professionals and researchers understand the impact of cognitive impairment on a person’s daily life	Reliable and accurate assessment of everyday functional abilities in older people. A recent study found that the ECog can detect early signs of neurodegenerative diseases, including Alzheimer’s, and track the progression of the disease	Farias et al., 2020
the Rey Auditory Verbal Learning Test (RAVLT)	An effective neuropsychological method for testing episodic memory that is frequently employed in dementia and pre-dementia cognitive assessments.	RAVLT is an effective early marker for detecting AD in people who have memory problems. However, RAVLT cannot be employed alone as screening tool, it is like one piece of the puzzle in evaluating cognitive impairment.	Moradi et al., 2017
the Functional Abilities Questionnaire (FAQ)	It measures the difficulties in ADLs, including self-care, mobility, communication, learning/applying knowledge, domestic life, community and civic life, and interpersonal interactions and relationships.	In clinical/research settings, the FAQ measures ADL concerns in a reliable and valid way. This test is best used to assess mild functional difficulties, which helps distinguish normal cognition from mild cognitive impairment and dementia. It has been found to have lower sensitivity than specificity.	González et al., 2022
Trail Making test (TMT-B)	the TMT-B can help assess the extent of cognitive decline and monitor changes over time. Performance on the TMT-B is timed, and the time taken to complete the task, along with any errors, can provide important information about cognitive functioning. Slower completion times or numerous errors may be indicative of cognitive impairment or executive dysfunction.	Other findings support TMT-B scores were not a significant predictor of AD progression. Accordingly, the results from TMT-B as diagnostic measures in research and as screening tools for SMC in clinical practice.	Papp et al., 2014; Warren et al., 2023

### Structural imaging

Structural imaging techniques, such as MRI, offer valuable clinical insights when examining the underlying factors contributing to cognitive decline (Harper et al., 2013). MRI is commonly performed to rule out other potential factors contributing to cognitive decline, rather than to confirm a diagnosis of AD (Frisoni et al., 2017). Structural MRI utilizes powerful magnets and radio waves to generate detailed brain images. It enables the measurement of brain tissue volume and the identification of structural alterations associated with AD. Alzheimer’s patients often exhibit atrophy, or shrinkage, in key brain regions, particularly the hippocampus and the entorhinal cortex, both critical for memory and learning (Vogel et al., 2019). MRI can detect Alzheimer’s by measuring brain tissue volume in these regions; a reduction in volume may indicate the disease. Additionally, MRI can identify Alzheimer’s by spotting changes in brain structure indicative of the presence of amyloid plaques and NFTs, the two key Alzheimer’s biomarkers. Trained radiologists use MRI to detect Alzheimer’s by observing signs such as atrophy in the hippocampus and entorhinal cortex, enlarged ventricles, white matter hyperintensities, microbleeds, amyloid plaques and neurofibrillary tangles.

PET is an imaging method that employs radioactive tracers to gauge the activity of specific molecules in the body. PET is instrumental in measuring the levels of Aβ and tau protein in the brain, both of which form plaques in the brains of individuals with AD (Zhang et al., 2021). Fludeoxyglucose positron emission tomography (FDG-PET) is another non-invasive imaging technique that employs a radioactive tracer called fluorodeoxyglucose (FDG) to measure glucose metabolism levels in the brain (Young et al., 2020). Glucose serves as the primary energy source for the brain, and FDG-PET assesses how efficiently the brain is functioning. FDG-PET is not advisable for diagnosing preclinical AD in patients due to the inability to determine if the hypometabolism is directly linked to AD pathology (Dubois et al., 2016). However, clinicians may consider referring patients with more pronounced symptoms for an FDG-PET scan to detect areas of glucose hypometabolism and neurodegeneration that may suggest AD (Frisoni et al., 2017).

## Confirming AD pathology

In the field of contemporary healthcare, there have been significant advancements in the confirmation of AD pathology (Hampel et al., 2018). AD, being a complex neurodegenerative condition, presents diagnostic challenges, underscoring the importance of early and precise detection. This multifaceted approach involves molecular investigations to identify genetic and protein markers, advanced imaging methods for observing structural and functional changes in the brain, and the examination of cerebrospinal fluid (CSF) for crucial biomarkers (Bader et al., 2020). Although it is understood that pathological changes commence prior to the manifestation of symptoms, it is challenging to ascertain if the presence of biomarkers indicating pathophysiological changes in the preclinical phase definitively indicates the development of clinical disease in an individual’s lifetime. Single biomarkers do not offer solid prognostic data. In recent times, there have been efforts to enhance the precision of diagnoses and the capability to anticipate individuals who are prone to experiencing clinical symptoms by considering a combination of biomarker discovery.

Jack and colleagues suggested that diagnosis should consider the presence and absence of the biomarkers categorized as amyloid, tau, and neurodegeneration (A/T/N) (Jack et al., 2016). This novel descriptive of ATN classification for AD has been recently developed to prioritize the pathological and physiological factors above traditional clinical measurements like cognitive test scores (Jack et al., 2016, 2018). In the ATN system, subjects are classified into three binary categories: amyloid burden, tau burden, and neurodegeneration. Each subject is assigned a rating of either normal (physiological, “−”) or abnormal (pathological, “+”). The resulting 8 groupings, each characterized by distinct combinations of biomarkers, span from A-T-N- (indicating the absence of pathology) to A+T+N+ (indicating the presence of pathology in all categories). There is a suggestion that any combination of ATN biomarkers with A+ indicates a pathogenic alteration associated with the AD continuum. Several recent research have investigated the potential of ATN to predict clinical progression and cognitive decline (Altomare et al., 2019; Jack et al., 2019; Soldan et al., 2019; van Maurik et al., 2019; Yu et al., 2019). In this subsection, diagnostic biomarker based on the ATN model, along with emerging biomarkers will be discussed.

## Biomarkers Aβ and pathologic tau (AT classification)

The biomarkers in the A+ group indicate the presence of aggregated Aβ (Baldeiras et al., 2022). Aβ peptides are produced through the enzymatic cleavage of APP by β- and gamma-secretases. While there are many isoforms of Aβ, almost 90% of the Aβ peptides present in the brain are either Aβ (Aβ1-40) or Aβ (Aβ1-42). Aβ1-42 constitutes the primary constituent of senile plaques. An increased abundance of senile plaques is essential for a neuropathological diagnosis of AD. Senile plaques can be detected through the use of cortical amyloid PET ligand binding (Beach, 2022). Additionally, cerebral Aβ aggregation can be identified by measuring Aβ1-42 and Aβ1-40 levels in CSF using non-radioactive, antibody-based techniques like ELISA (Camporesi, 2021). An inherent trait of early AD is a decrease in CSF levels of Aβ1-42, likely caused by the accumulation of the peptide in senile plaques. However, certain studies have indicated that the ratio of CSF Aβ (Aβ1-42)/(Aβ1-40) may serve as a more reliable measure of Aβ production and aggregation, as opposed to solely examining Aβ1-42 levels (Niemantsverdriet et al., 2017).

The biomarkers observed in the T+ group indicate the presence of aggregated Tau (Baldeiras et al., 2022). Tau proteins are soluble microtubule-associated proteins (MAPs) that strongly stabilize axonal microtubules. Tau undergoes hyperphosphorylation in AD, resulting in its detachment from microtubules (Alonso et al., 2018). Unconstrained, excessively phosphorylated Tau is susceptible to enzymatic breakdown, as well as self-assembly into harmful clusters and ultimately forming paired helical filaments (PHFs) and NFTs. Cortical Tau PET ligand binding can detect aggregated Tau, particularly PHFs (Okamura et al., 2018). Nevertheless, research has demonstrated that the presence of phosphorylated Tau in CSF is indicative of Tau disease (Wattmo et al., 2020). Neurons that have NFTs emit phosphorylated Tau, which can be quantified in the CSF by antibody-based immunoassays. More than 40 locations on Tau have been demonstrated to undergo phosphorylation in AD; yet Tau phosphorylated at threonine 181 (pTau181) is among the most extensively studied phosphorylated Tau indicators (Suárez-Calvet et al., 2020). Studies have demonstrated that CSF levels of pTau181 are increased in persons with AD and are strongly associated with the extent of Tau pathology observed after death (Shoji, 2019; Thijssen et al., 2020). Furthermore, this biomarker has demonstrated a high level of specificity for AD since elevated levels of CSF pTau181 are not observed in other tauopathies. The investigation of phosphorylated Tau at serine 199 (pTau199) and threonine 231 (pTau231) as possible biomarkers is ongoing (Lewczuk et al., 2004). The levels of pTau199 and pTau231 in the CSF are strongly associated with pTau181 CSF levels and demonstrate comparable diagnostic accuracy (Shoji, 2019). Apart from that, cross-sectional studies covering the entire clinical AD continuum have revealed that plasma isoforms p-tau181 and p-tau217 may distinguish amyloid-PET or tau-PET positive cases from amyloid-PET or tau-PET negative cases. These cross-sectional investigations have also demonstrated that plasma p-tau levels can identify patients with AD dementia from those with frontotemporal lobar degeneration (Janelidze et al., 2020; Karikari et al., 2020; Palmqvist et al., 2020; Thijssen et al., 2020). Mattsson-Carlgren et al. (2020) enrolled 250 non-demented participants from the Swedish BioFINDER study and employed the “Meso Scale Discovery” (MSD) Eli Lilly immunoassay to quantify p-tau217 levels at baseline and during follow-up. The findings revealed that patients in the preclinical and early clinical stages of AD have higher levels of p-tau217 than cognitively healthy controls. Furthermore, higher p-tau217 levels were linked to an increased likelihood of developing AD dementia, as well as faster rates of cognitive decline and thinning of the temporal cortex and hippocampus (Mattsson-Carlgren et al., 2020).

**FIGURE 3:**
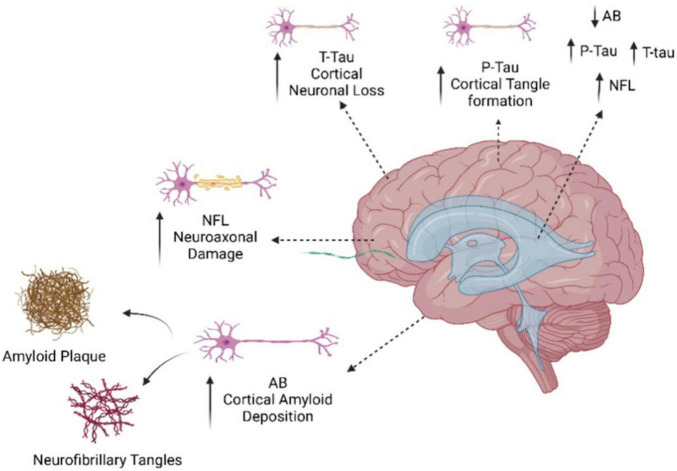
Illustration of neurodegenerative deterioration in the brain and associated indicators. As portrayed, brain damage could be caused by the deposition of amyloid beta protein in the brain, resulting in amyloid plaques, as well as the creation of neurofibrillary tangles among neurons. These alterations cause the loss of neurons in the cortex, the brain’s outer layer. The results imply that brain traumas sustained in the NfL may raise the incidence of AD.

Recently, many studies have shown that CSF p-tau along with Aβ42, and t-tau together are the key biomarkers for AD. For instance, Suárez-Calvet et al. (2020) reported CSF p-tau is major prognosis marker in AD as it distinguishes dementia associated with AD from cognitively unimpaired (CU) and MCI, also CSF p-tau is useful for disease staging (Suárez-Calvet et al., 2020). Furthermore, the biomarker’s robustness and reliability as an early diagnosis tool for AD is enhanced by the fact that Aβ plaque formation occurs years, if not decades, prior to the onset of symptoms (Bălaşa et al., 2020). The measurement of Aβ and tau proteins in CSF continued to be a focus till now. In another study done by Teunissen et al. (2018) have been included an Aβ42 as standard AD diagnostic guideline (Teunissen et al., 2018). It has been shown that a fluctuation in CSF Aβ42 level occurs 10–20 years before the beginning of visible symptoms which make it helpful tool (Teunissen et al., 2018). Moreover, in the same study showing that Aβ42 biomarker concentration differs in plasma from CSF that showed reduced levels in CSF compared to plasma high levels (Teunissen et al., 2018). This could be due to the blood-brain barrier (BBB) accessibility and transportation of all biomolecules, and high levels of Aβ42 found in plasma due to avoiding accumulating Aβ42 in in the brain (clearance system). In addition, integrating the Aβ42/Aβ40 and Aβ42/Aβ38 ratios with T-tau and P-tau levels is likely the most advantageous method to develop a diagnostic tool based on these two biomarkers. This technique offers a sensitivity and specificity of approximately 85–95% (Jin et al., 2019). Recently, blood-based biomarkers for AD, like tau and Aβ, have been incorporated by Rissman et al. (2024) into screening algorithms in an attempt to increase screening precision. They used plasma samples from the first group of participants screened for AHEAD and used immunoprecipitation liquid chromatography-tandem mass spectrometry (LC-MS/MS) to measure phosphorylated and non-phosphorylated forms of tau181 and tau217 alongside Aβ42 and Aβ40 in order to further validate plasma p-tau species as early AD biomarkers. This work aimed to predict brain amyloid PET status in cognitively unimpaired patients using MS measured plasma p-tau217, np-tau217, p-tau181, concentration ratios, and Aβ42/Aβ40 ratio data. The results show improved performance for the identification of amyloid PET positive cognitively unimpaired individuals using plasma p-tau217/np-tau217; however, the combination of plasma p-tau217 and Aβ42/Aβ40 ratios in a model that predicted cerebral amyloid PET status yielded the best performance, as indicated by AUC (Rissman et al., 2024). Figure 3 depicts the process of neurodegenerative decline in the brain and the corresponding markers that are connected with it.

## Biomarkers of neurodegeneration or neuronal injury (N classification)

The biomarkers observed in the N+ group indicate the presence of neurodegeneration. Axonal degeneration is a prominent characteristic of AD and is more strongly associated with the beginning of cognitive impairment compared to other clinical aspects. Neurodegeneration in brains affected by AD can be identified by the use of FDG PET hypometabolism and MRI. Nevertheless, studies have demonstrated that persons with AD exhibit elevated levels of t-tau in the CSF, and these levels are strongly associated with the extent of neurodegeneration. However, neurodegeneration is not exclusive to AD and can be observed in various other illnesses affecting the neurological system. Nevertheless, when employed alongside other indicators, t-tau can offer crucial insights into an individual’s placement on the AD spectrum and the extent of their cognitive decline (Alcolea et al., 2021).

## Other promising biomarkers

### Blood-based approaches

Blood, in contrast to CSF, which requires lumbar puncture for accessibility, comprises less invasive body fluids and is readily accessible for the purposes of diagnosing, evaluating, and monitoring the progression of AD (Villa et al., 2020). The Alzheimer’s Association recommends that specialized memory clinics may employ blood biomarkers to aid in diagnosing patients with cognitive impairment. Several blood biomarkers, such as plasma Aβ42, Aβ42/40 ratio, p-tau, t-tau, neurofilament light polypeptide (NfL), glial fibrillary acidic protein (GFAP), and soluble triggering receptor expressed on myeloid cells 2 (sTREM2), have been identified as potential biomarkers for AD (Tao et al., 2023). However, despite significant research activity, a complete and up-to-date summary of the key blood-based biomarker candidates remains insufficient.

Early investigations employed enzyme linked immunosorbent assays (ELISA) immunoassays to evaluate the concentration of Aβ40 and Aβ42 in plasma as predictors of conversion to AD in patients with MCI. In this investigation, the plasma samples were obtained at baseline from two independent cohorts of patients with MCI and age-matched controls (prodromal stage). The results demonstrated a negative correlation with AD and the authors concluded that the CSF biomarkers are better predictors of progression to AD than plasma Aβ isoforms (Hansson et al., 2010). Following this, a study utilizing single-molecule array (Simoa) was conducted to analyze plasma levels of Aβ42 and Aβ40 in a cohort of 719 individuals, including patients with subjective cognitive decline (SCD), MCI, AD dementia and cognitively healthy elderly. Results revealed a decrease in plasma Aβ42 concentration in individuals with AD compared to the control group. This study concluded that during the dementia stage of AD, plasma Aβ is markedly reduced, suggesting that significant alterations in Aβ metabolism take place later in the peripheral rather than in the brain (Janelidze et al., 2016). Currently, there is insufficient data to support the use of plasma Aβ42/40 as a reliable method for distinguishing between AD and other forms of dementia. Given their limited availability and somewhat high cost, both Simoa and IP/MS based assays require further optimization in several aspects before they may be effectively employed for screening AD in large populations.

### Saliva

In addition to the CSF, several emerging biomarkers from tears and saliva, are being found to predict AD. The non-invasive, convenient, and cost-effective collection of saliva makes it an attractive marker for monitoring diseases. Besides that, CSF shows a relationship with saliva which proteins are secreted into saliva (Ashton et al., 2019). The diagnostic performance of AD-specific salivary biomarkers has been included Aβ1-40, Aβ1-42, p-tau, t-tau and lactoferrin in many research and studies (Pawlik and Błochowiak, 2021). Aβ levels were found and deposited in many body tissues including nasal mucosa, skin, and other gland, in addition to its main build up in the brain. Moreover, APP and Aβ are the mostly expressed in epithelia cells in saliva (Pawlik and Błochowiak, 2021). Saliva Aβ1-42 levels biomarkers are specific as they can differentiate patients with AD, but not patients with other neurological disease such as Parkinson’s disease (PD). More importantly, it can be used to diagnose early stages of the disease, cognitive difficulties, the severity and progression of AD, and not merely as an approach of identifying AD, but to distinguish it from other neurodegenerative diseases (Pawlik and Błochowiak, 2021). Other than Aβ salivary biomarkers which is abundant in salivary, lactoferrin also shown to have Aβ-binding properties and thus could play an important role in the pathophysiology of AD (Farah et al., 2018). Although saliva can serve as a valuable source of markers, its composition may be influenced by factors such as the circadian cycle, flow rate, and timing of sample collection (Farah et al., 2018). In addition, the presence of degradative enzymes leads to the instability of biomarker levels, necessitating the process of normalization.

### Tears

The eyes have a close relationship with the brain, which considers tears as a potential source of biomarker for AD. And interestingly, the presence of Aβ plaques and tau deposits in the retina and lens has been recognized at the cellular level. Moreover, certain investigations have demonstrated a correlation between the accumulation of protein deposits in the eyes of individuals with AD and the formation of such deposits in the brain (Kaštelan et al., 2023). The discovery of potential AD biomarkers in tear samples could be exceptionally useful for conducting screenings among the general public (Majeed et al., 2021). Del Prete et al. (2021) conducted a study where they found increased quantities of Aβ42 protein in the tears of two healthy persons with a family history of AD (pre-clinical stage). This was determined using an immunocytochemistry technique (Del Prete et al., 2021). The study discovered a clear correlation between the presence of Aβ42 in tears and the development of retinal plaques. This correlation was not observed in the tear samples of a healthy participant without a family history of the condition. Given that the individuals being studied exhibited no apparent clinical symptoms of AD, the discovery of Aβ42 in tear samples has the potential to be utilized for early Alzheimer’s diagnosis and for screening purposes. Gharbiya et al. (2023) conducted an analysis of the amounts of Aβ peptide Aβ1-42, the C-terminal fragment of amyloid precursor protein (APP-CTF), and p-tau in the tears of individuals with MCI, mild to severe AD, and healthy volunteers (Gharbiya et al., 2023). Their investigation demonstrated that the concentration of tears Aβ1-42 could effectively distinguish both MCI and AD patients with a high degree of specificity (93%) and sensitivity (81%). Moreover, the study found no significant variations in the abundance of APP-CTF and p-tau in tear samples. As per their findings, assessing the levels of Aβ1-42 in tears could offer a minimally invasive approach for the early detection and diagnosis of AD. The presence of reduced Aβ1-42 levels in tears may represent a specific, sensitive, non-invasive, and cost-effective biomarker for the early identification of AD. More importantly, tears biomarkers hold great promise for enhancing diagnostic precision, tracking disease advancement, and assessing the effectiveness of treatments. Also, they are easily accessible, non-invasive, less costly compared with other diagnostic tools, and can be performed by healthcare practitioners without the need for specialized training (Chaitanuwong et al., 2023).

### MicroRNAs (miRNAs)

Another genetic potential biomarker for AD is miRNA, a small non-coding RNA molecules that play a role in regulating gene expression (Nikolac Perkovic et al., 2021). A miRNA is a single-stranded RNA that is 19 to 24 nucleotides long and plays a major role in post-transcriptional gene silencing. Also, it is a very effective tool in early diagnosis of AD since miRNAs has been investigated as marker of AD pathogenesis (Nikolac Perkovic et al., 2021). A wide range of peripheral circulation (serum, plasma, exosomes, whole blood, peripheral blood mononuclear cells) and CSF miRNAs are commonly detected. More importantly, brain tissue has been linked to non-circulating miRNAs (Nikolac Perkovic et al., 2021). Accordingly, in a study by Zhang et al. (2019), a meta-analysis of ten different studies illustrated that miRNA as an AD diagnostic biomarker have overall and diagnostic odds ratio of 14 (95% CI: 11–19) sensitivity 0.80 (95% CI: 0.75–0.83) and specificity 0.83 (95% CI: 0.78–0.86) which represents an accurate and reliable biomarker (Zhang et al., 2019). Furthermore, miRNAs are highly promising indicators for diagnosing diseases. More recently, studies have been conducted on diagnostic efficiency and accuracy on AD patients and can be characterized healthy people from AD (Hu et al., 2016; Lusardi et al., 2017; Zhao et al., 2020).

## Pharmacological approach

### FDA-approved drugs

Prior to recent developments, patients with AD had access to only symptomatic treatments, such as acetylcholinesterase inhibitors. The most recent addition to this class of drugs is galantamine, which was approved by the US Food and Drug Administration (FDA) in 2001 (Cronin, 2001). Another treatment option is memantine, a noncompetitive N-methyl-D-aspartate receptor antagonist, which received FDA approval in 2003 (Zarotsky et al., 2003).

Acetylcholinesterase (AChE) inhibitors are employed for patients with mild cognitive impairment or mild dementia stage disease (stage 4 based on FDA classification) to impede ACh degradation and, in turn, boost neural cell function by increasing ACh levels (Akıncıoğlu and Gülçin, 2020). The cholinergic theory has garnered significant attention and has been the subject of much research, leading to the development of three authorized drugs for the treatment of AD. Tacrine, a type of medication known as a cholinesterase inhibitor (ChEI), was initially granted approval by the FDA as the first therapy for the treatment of AD. However, its administration was subsequently terminated due to the adverse effects it posed on liver function, known as hepatotoxicity. At present, the three ChEIs employed in the therapeutic management of people with AD are donepezil, rivastigmine and galantamine (Liu et al., 2019) Figure 4 shows current treatments involving ChEIs with mechanism. In general, ChEIs are commonly perceived to possess poor therapeutic efficacy and are primarily acknowledged for their moderate capacity in managing the symptoms associated with AD (Kepp, 2012). However, additional evidence is being uncovered that suggests a more intricate mechanism underneath the cholinergic system. This mechanism has the ability to interact with other pathological aspects of AD, such as aberrant Aβ and tau cascade, inflammation, apoptosis, and imbalances in neurotransmitter and neurohormonal systems (Wang and Zhang, 2018).

**FIGURE 4:**
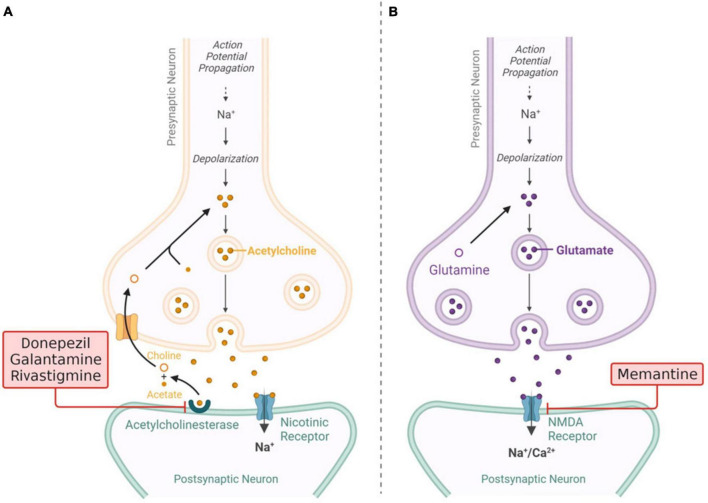
**(A)** Visualization of current drugs (donepezil, galantamine, rivastigmine) **(B)** mechanism of action of memantine for Alzheimer’s disease (AD). Adaptation from Breijyeh and Karaman (2020).

**FIGURE 5:**
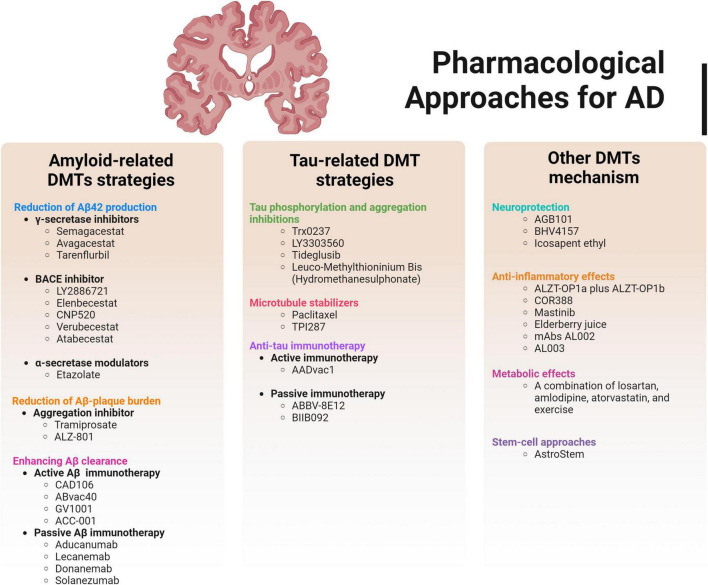
Summary of pharmacological approaches for AD involving amyloid-related DMTs strategies, tau-related DMT strategies and other DMTs mechanisms.

Memantine is an uncompetitive and NMDA receptor antagonist that is approved for the treatment of moderate to severe AD (stage 5 and 6 based on FDA classification). Memantine modulates glutamate activity, preventing excessive stimulation that can lead to neuronal damage (Figure 5). This drug can help manage symptoms and slow cognitive decline in later stages of the disease (Schmidt, 2022). Even so, it should be noted that memantine exhibits restricted clinical effectiveness (Matsunaga et al., 2015). Given this perspective, there is a notable interest in exploring novel moderate-affinity NMDAR antagonists that possess similar yet distinguishable pharmacological characteristics. In the recent past, a new polycyclic amine called RL-208 has been synthesized (Companys-Alemany et al., 2020). This compound acts as a voltage-dependent, moderate-affinity, uncompetitive blocker of NMDA receptors. Its pharmacological and electrophysiological properties have been thoroughly investigated using *in vitro* methods (Companys-Alemany et al., 2020). However, memantine has a number of potential adverse effects. Common side effects include headaches, dizziness, elevated blood pressure, drowsiness, restlessness, and hallucinations. Less often occurring side effects include asthenia, constriction, diarrhea, nausea, anorexia, coughing, and breathing problems (Shafiei-Irannejad et al., 2021).

Over the course of nearly two decades, despite multiple clinical studies, the prospects for advancing novel therapy were desolate and discouraging (Cummings et al., 2022). In June 2021, the FDA granted expedited approval to aducanumab (AduhelmTM), a monoclonal antibody (mAb) called anti-amyloid-β (Aβ) that specifically targets “protofibrils” in patients who have MCI-AD or who are in the mild dementia stage of the disease (stage 3 and stage 4 based on FDA classification). These protofibrils were first characterized in the 1990s by Walsh et al. (1997) and have since been recognized as important neurotoxins. Aducanumab has obtained the initial approval as a drug that targets the fundamental cause of AD, despite the presence of significant negative consequences. The FDA clearance sparked significant controversy due to the adverse effects such as brain swelling, small brain bleeding, headache and falls, as well as limited effectiveness data (Kuller and Lopez, 2021).

In January 2023, the FDA granted expedited approval to lecanemab (LeqembiTM), a monoclonal antibody that targets anti-Aβ protofibrils, for its similar mechanism of action and side effects (stage 3 and stage 4 based on FDA classification). However, this time, there was less controversy surrounding the approval due to the clinical trial data clearly showing a reduction in the progression of memory loss (Van Dyck et al., 2023). The FDA’s Accelerated Approval Program provides support for medications that effectively treat severe medical problems and demonstrate a predictive marker indicating clinical benefit. This approach expedites the process of bringing a medicine to market compared to the conventional approval method, however, it relies on predicting rather than demonstrating the clinical advantages. In July 2023, the FDA awarded full authorisation to lecanemab for the treatment of early-stage AD after conducting further examination. Lecanemab and aducanumab effectively eliminate toxic Aβ protofibrils from the brain affected by AD. However, their usage is associated with notable adverse effects known as amyloid-related imaging abnormalities (ARIA), which may potentially induce symptoms such as headaches, exacerbation of cognitive impairment, dizziness, visual impairment, nausea, and seizures. Furthermore, a meta-analysis of clinical studies investigating possible treatments for AD, such as aducanumab, lecanemab, and donanemab, discovered that monoclonal antibodies (mAbs) that produce ARIA may lead to an increased rate of brain shrinkage (Alves et al., 2023). Hence, the ongoing struggle against AD persists, necessitating patients and their caregivers to meticulously evaluate the advantages and disadvantages of these treatments.

Recently, the FDA has granted approval for the TRAILBLAZER-ALZ 2 Phase 3 study (donanemab-azbt, 350 mg/20 mL once-monthly injection for IV infusion) on 2 July 2024. This drug will be used to treat persons with early symptoms of Alzheimer’s disease, including those with moderate cognitive impairment (MCI) and mild dementia with confirmed amyloid plaques (Wall, 2024). Those with a reduced risk of disease progression had the best outcomes with Kisunla in the TRAILBLAZER-ALZ 2 Phase 3 trial. Over the course of 18 months, trial participants were divided into two groups for analysis: the general population, which also included individuals with high tau levels, and a group of patients who were less advanced in their disease and had low to medium amounts of tau protein. In both groups, Kisunla treatment markedly reduced clinical deterioration. Those with less advanced disease who received treatment with Kisunla had a noteworthy 35% reduction in cognitive decline when compared to placebo on the integrated Alzheimer’s Disease Rating Scale (iADRS), which evaluates thinking, memory, and day-to-day functioning. Additionally, employing statistical significance, the response to treatment was observed in the entire population (Bucci et al., 2021; Wall, 2024).

## Present state of the landscape treatment

Ongoing research is primarily dedicated to the advancement of therapeutic strategies aimed at decelerating or halting the progression of the disease. This research considers the latest findings in the disease’s biology, diagnostic markers, accurate diagnosis of each individual’s disease state, and the design of clinical trials. Moreover, drug development research for AD has become increasingly complex due to the potential inclusion of preclinical and prodromal AD populations in current trials, in addition to the previously included groups representing all clinical phases of AD dementia (Dubois et al., 2016). Molecular targets for treating AD are typically involved in Aβ or p-tau synthesis, as well as Aβ plaque and NFT development. The toxic proteinopathy theory implies that Aβ plays a role in a gain-of-function process. As Aβ deposition is linked to AD degenerative changes, reducing Aβ levels could prevent neurodegeneration and cognitive loss (Ezzat et al., 2023). However, despite decades of research, the failing findings of current therapeutic studies aimed at counteracting Aβ formation or favoring Aβ clearance prompt a critical evaluation of the amyloid cascade concept (Granzotto and Sensi, 2023). The primary objection to designating the Aβ pathway as the initiator of neurodegeneration is related to data showing that Aβ deposits are not predominantly correlated with cognitive function, that Aβ deposits can be found in people with normal cognitive function, and that neuronal injury and tau pathology markers can exist independently of Aβ deposition (Perez-Nievas et al., 2013). The theory of a protein loss-of-function has been established in contrast to the gain-of-function mechanism, and it is likewise supported by translational and genetic investigations (Ezzat et al., 2023).

The development of Aβ aggregates in the brain suggests a mechanism that goes beyond protein accumulation: the depletion of proteins in fluid. Since several studies have shown that Aβ-42 low CSF levels are associated with the longitudinal development of AD symptoms and with neurodegenerative markers, and that low Aβ levels better correlate with cognitive decline than the burden of the insoluble form, it is also possible to argue that the depletion of Aβ soluble forms is a crucial mechanism in neurodegeneration (Villemagne and Chételat, 2016; McDade et al., 2018). The findings that both sporadic and hereditary types of AD are associated with normal cognition and high levels of soluble Aβ-42 in brain aberrant amyloid burden supports the loss-of-function hypothesis (Sturchio et al., 2021, 2022).

The unsatisfactory outcomes of anti-amyloid therapy strategies can be partially explained by the intricacy of the implicated pathways and the poor understanding of the amyloid cascade and its effects. There is substantial evidence to suggest that the primary toxic Aβ species in AD are oligomers (Rinauro et al., 2024). The amount of soluble Aβ is correlated with the severity of neurodegenerative alterations rather than the burden of senile plaques, and oligomers are cytotoxic and break down synapses *in vitro* (Kreiser et al., 2020). Targeting plaques, fibrils, protofibrils, and oligomers hence suggests more variation in the therapeutic response. Furthermore, the “amyloid cascade” is only one among numerous molecular modifications that define AD, including tau-mediated toxicity and neuroinflammation, and it starts decades before the onset of symptoms. Therefore, it’s possible that the anti-Aβ treatment strategies currently in practice will be insufficient to prevent AD (Zhang et al., 2023).

This section will provide a discussion of the drugs that are currently being explored as potential disease-modifying therapies (DMTs). Additionally, it will briefly cover the ongoing clinical trials in AD that are in phases 1, 2, and 3 that currently being presented in the official clinical trial website (clinicaltrials.gov). However, the limitation of these studies were the ambiguity and lack of the study outcomes for certain intervention presented in the website. Due to these gaps, we couldn’t specify the projection of the completed study interventions as either being approved for further investigation or merely for research purpose. Figure 5 shows the overall summary of the pharmacological approaches for AD.

## Current AD DMT research

Most molecules investigated as possible targets for AD-modifying therapy are involved in the formation of Aβ plaque and NFT, as well as in the generation of Aβ or p-tau (Tondo et al., 2024).

### Amyloid-related DMTs strategies

Anti-amyloid DMTs have primarily targeted three major mechanisms of action (MOAs): (i) decreasing the production of Aβ42 (through the use of γ-secretase inhibitors, β-secretase inhibitors, or α-secretase potentiation), (ii) reducing the accumulation of Aβ plaques (by employing aggregation inhibitors or drugs that interfere with metals), and (iii) enhancing the clearance of Aβ (via active or passive immunotherapy) (Livingston et al., 2019). Table 4 summarized current clinical trial status employing all DMTs strategies including Aβ, tau and other mechanisms contribute to AD (clinicaltrials.gov).

**TABLE 4:** Current clinical status of amyloid-related DMTs strategies, tau-related DMTs strategies and DMTs of other mechanisms.

Drug	Therapy type and purpose	Identifier	Sponsor	Clinical phase	Status/result outcomes
**Amyloid-related DMTs strategies**					
**1. Reduction of Aβ 42 production**					
Semagacestat	• γ-secretase inhibitors • To assess the safety of semagacestat in AD patients during 24 months of open-label treatment	NCT01035138	Eli Lilly and Company	Phase 3	Study was terminated in 2011 as Semagacestat did not slow disease progression and was associated with worsening of clinical measures of cognition and the ability to perform activities of daily living.
Semagacestat (LY450139)	• γ-secretase inhibitors • To measure the effect of semagacestat on both β-amyloid and amyloid plaques for some patients.	NCT00762411	Eli Lilly and Company	Phase 3	Study was terminated in 2011 as Semagacestat did not slow disease progression and was associated with worsening of clinical measures of cognition and the ability to perform activities of daily living.
Avagacestat (BMS-708163)	• γ-secretase inhibitors • The purpose of this study is to determine the safety and tolerability of BMS-708163 in patients with mild to moderate AD over a treatment period of 12-weeks and the course of any potential effects during a 12-week wash-out period	NCT00810147	Bristol-Myers Squibb	Phase 2	Study was completed in 2010. Avagacestat dosed at 25 and 50 mg daily was relatively well tolerated and had low discontinuation rates. The 100-mg and 125-mg dose arms were poorly tolerated with trends for cognitive worsening. This study establishes an acceptable safety and tolerability dose range for future avagacestat studies in AD (Coric et al., 2015).
Tarenflurbil (MPC-7869)	• γ-secretase inhibitors • To determine the efficacy, safety, and tolerability of tarenflurbil.	NCT00105547	Myrexis Inc.	Phase 3	Study was completed in 2008 and the outcome showed that Tarenflurbil did not slow cognitive decline or the loss of activities of daily living in patients with mild AD (Green et al., 2009).
Tarenflurbil (MPC-7869)	• γ-secretase inhibitors • To evaluate the safety and efficacy of 800 mg twice daily MPC-7869 compared to placebo and to assess the effects of daily treatment on cognition, ADLs, and global function in mild AD patients.	NCT00322036	Myrexis Inc	Phase 3	Terminated (2008)
LY2886721	• BACE inhibitors • To assess individuals with MCI related to AD or mild AD and amyloid plaque-positive subjects’ drug responsiveness.	NCT01561430	Eli Lilly and Company	Phase 1/2	Terminated in 2018 due to abnormal liver biochemical tests in some participants.
Elenbecestat (E2609)	• BACE inhibitors • To evaluate the efficacy and safety of Elenbecestat (E2609) in subjects with early AD	NCT02956486	Eisai Co., Ltd.	Phase 3	Terminated in 2021 due to no evidence of potential efficacy, and the adverse event profile of E2609 being worse than placebo
Verubecestat (MK-8931)	• BACE inhibitors • To assess MK-8931’s safety and effectiveness in prodromal AD patients with amnestic MCI	NCT01953601	Merck Sharp & Dohme LLC	Phase 3	Terminated (2019)
Atabecestat	• BACE inhibitors • To assess whether atabecestat slows cognitive decline compared to placebo, as measured by the Preclinical Alzheimer Cognitive Composite (PACC), in amyloid-positive, asymptomatic Alzheimer’s risk participants.	NCT02569398	Janssen Research & Development, LLC	Phase 2 and 3	Terminated in 2020 due to change in benefit-risk profile for individuals with early sporadic AD owing to elevations in liver enzymes in subjects receiving atabecestat
Etazolate (EHT 0202)	• α-secretase modulators • To compare the safety and tolerability of two doses of EHT 0202 (40 mg and 80 mg b.i.d) versus placebo, as well as the exploratory efficacy of acetylcholinesterase inhibitor on cognition, behavior, activities of daily living, caregiver burden, and patient global assessment over 3 months.	NCT00880412	Exonhit	Phase 2	Study was completed in 2009, however, the study results have not been submitted in clinical trial website
**Amyloid-related strategies**					
**2. Reduction of Aβ -plaque burden**					
ALZ-801	• Aggregation inhibitor • To evaluate the pharmacokinetics of ALZ-801, tramiprosate, and its major metabolite, NRM5074, in prototype drug product formulations and the influence of food on the prototype tablet formulation’s bioavailability.	NCT04585347	Alzheon Inc	Phase 1	Study was completed in 2015. ALZ-801 was well tolerated and there were no severe or serious adverse events (AEs) or laboratory findings. A clinical dose of ALZ-801 (265 mg twice daily) was established that achieves the AUC exposure of 150 mg of tramiprosate twice daily, which showed positive cognitive and functional improvements in apolipoprotein E4/4 homozygous AD patients (Hey et al., 2018).
	• Aggregation inhibitor • The study will examine how oral ALZ-801 affects core AD pathology biomarkers in Early AD patients with the APOE4/4 or APOE3/4 genotype.	NCT04693520	Alzheon Inc.	Phase 2	Active, not recruiting (2024)
	• Aggregation inhibitor • To evaluate the safety and efficacy of ALZ-801 in Early Alzheimer’s disease (AD) subjects with the APOE4/4 genotype.	NCT04770220	Alzheon Inc.	Phase 3	Active, not recruiting (2024)
**Amyloid-related strategies**					
**3. Enhancing Aβ clearance**					
CAD106 and CNP520	• Active Aβ immunotherapy • To assess if CAD106 and CNP520, given separately, could reduce the onset and progression of AD clinical symptoms in people at risk due to age and genotype.	NCT02565511	Novartis Pharmaceuticals	Phase 2 and 3	Terminated (2021)
CAD106	• Active Aβ immunotherapy • To assess safety, tolerability, and abeta-specific antibody response after repeated i.m., adjuvanted CAD106 injections	NCT01097096	Novartis Pharmaceuticals	Phase 2	Study was completed in 2012, however, the study results have not been submitted in clinical trial website
ABvac40	• Active Aβ immunotherapy • To assess tolerability and safety of repeated subcutaneous administration of ABvac40 in patients with mild to moderate AD.	NCT03113812	Araclon Biotech S.L.	Phase 1	Study was completed in 2015. The study concluded that ABvac40 showed a favorable safety and tolerability profile while eliciting a consistent and specific immune response (Lacosta et al., 2018).
	• Active Aβ immunotherapy • The goal of this Phase II study is to demonstrate in people with a-MCI or vm-AD the same level of safety and tolerability found in the Phase I clinical trial of ABvac40 in people with mm-AD. It is also to evaluate the immune reaction that ABvac40 elicits and how it affects biomarkers for AD.	NCT03461276	Araclon Biotech S.L.	Phase 2	Study was completed in 2023, however, further outcomes of the study were not mentioned in the clinical trial website.
GV1001	• Active Aβ immunotherapy • To evaluate the efficacy and safety of donepezil and combined with GV1001 in Alzheimer patients	NCT03184467	GemVax & Kael	Phase 2	Study was completed in 2019. The results indicate that GV1001 1.12 mg met the primary endpoint of a statistically significant difference. GV1001 was well tolerated without safety concerns. This study warrants a larger clinical trial (Koh et al., 2021).
ACC-001	• Active Aβ immunotherapy • To determine safety, tolerability, and immunogenicity of ACC-001 with qs-21 adjuvant in subjects with mild to moderate AD	NCT00955409	Pfizer	Phase 2A	Study was completed in 2013. In 2013 the sponsor decided that ACC-001 would not be further developed in mild to moderate AD, study drug administration was discontinued, and remaining participants were followed for safety for up to 6 months after last injection.
Aducanumab (Aduhelm)	• Passive Aβ immunotherapy • To determine if aducanumab is safe and well tolerated following 100 weeks of treatment after a wash-out period caused by the end of feeder studies in people who had previously received aducanumab (i.e., previously treated participants) or a placebo (i.e., treatment-naïve participants).	NCT04241068	Biogen	Phase 3	Active, not recruiting (2024)
Lecanemab	• Passive Aβ immunotherapy • To determine if lecanemab is safe, well-tolerated, and effective in people with early AD	NCT01767311	Eisai Inc.	Phase 2	Active, not recruiting (estimation completed 2025)
	• Passive Aβ immunotherapy • This study examines if lecanemab is safe and well tolerated over the long term in people with EAD who are in the Extension Phase. It also checks to see if the long-term benefits of lecanemab, as measured by the CDR-SB at the end of the Core Study, are still present in the Extension Phase.	NCT03887455	Eisai Inc.	Phase 3	Active, not recruiting (estimation completed 2027)
Donanemab (LY3002813) (TRAILBLAZER-ALZ 3)	• Passive Aβ immunotherapy • To evaluate the safety and efficacy of donanemab in participants with preclinical AD.	NCT05026866	Eli Lilly and Company	Phase 3	Recruiting (estimation completed 2027)
Donanemab (LY3002813) (TRAILBLAZER-ALZ 5)	• Passive Aβ immunotherapy • To assess the safety and efficacy of donanemab in participants with early AD.	NCT05508789	Eli Lilly and Company	Phase 3	Recruiting (estimation completed 2027)
Donanemab (LY3002813) (TRAILBLAZER-ALZ 6)	• Passive Aβ immunotherapy • To investigate different donanemab dosing regimens and their effect on the frequency and severity of ARIA-E in adults with early symptomatic AD and explore participant characteristics that might predict risk of ARIA.	NCT05738486	Eli Lilly and Company	Phase 3	Recruiting (estimation completed 2025)
Solanezumab (LY2062430)	• Passive Aβ immunotherapy • To investigate the safety and efficacy of the study drug solanezumab in participants with prodromal AD.	NCT02760602	Eli Lilly and Company	Phase 3	Terminated in 2018 due to insufficient scientific evidence that solanezumab would likely demonstrate a meaningful benefit to participants with prodromal AD as defined by study protocol.
Solanezumab	• Passive Aβ immunotherapy • To determine if solanezumab will slow down the cognitive decline of AD compared to a placebo in people who already have mild AD.	NCT01900665	Eli Lilly and Company	Phase 3	Terminated in 2018 due to Solanezumab did not meet the study’s primary endpoint.
Solanezumab	• Passive Aβ immunotherapy • This is an open-label extension study in Alzheimer’s patients who have completed participation in either solanezumab Clinical Trial H8A-MC-LZAM (NCT00905372) or H8A-MC-LZAN (NCT00904683).	NCT01127633	Eli Lilly and Company	Phase 3	Terminated in 2018 due to Solanezumab did not meet the primary endpoint in study H8A-MC-LZAX.
Solanezumab (DIAN-TU)	• Passive Aβ immunotherapy • To evaluate the safety, tolerability, biomarker, cognitive, and clinical efficacy of investigational products in AD patients with a mutation by examining if the drug slows cognitive/clinical impairment or improves biomarkers.	NCT01760005	Washington University School of Medicine	Phase 3	Recruiting (estimation completed 2027)
ALZ-801 (APOLLOE4)	• Passive Aβ immunotherapy • To evaluate the safety and efficacy of ALZ-801 in Early AD subjects with the APOE4/4 genotype	NCT04770220	Alzheon Inc.	Phase 3	Active, not recruiting (estimation completed 2024)
ALZ-801 (APOLLOE4)	• Passive Aβ immunotherapy • To investigate the effects of oral ALZ-801, in subjects with Early AD who have the APOE4/4 or APOE3/4 genotype, on the biomarkers of core AD pathology	NCT04693520	Alzheon Inc.	Phase 2	Active, not recruiting (estimation completed 2024)
ABBV-916	• Passive Aβ immunotherapy • To assess safety of ABBV-916 and how intravenous ABBV-916 moves through body and affects brain amyloid plaque clearance in adult participants (Aged 50–90 years) with early AD	NCT05291234	AbbVie	Phase 2	Recruiting (estimation completed 2030)
**Tau-related DMTs strategies**					
TRx0237	• Aggregation inhibitor • To compare TRx0237 16 mg/day and 8 mg/day to placebo in AD therapy. To prove TRx0237’s disease-modifying efficacy, an open-label, delayed-start phase is included.	NCT03446001	TauRx Therapeutics Ltd	Phase 3	Study was completed in 2023, however, the study results have not been submitted in clinical trial website
LY3303560	• Phosphorylation inhibitor • To evaluate the safety, tolerability, and pharmacokinetics in healthy subjects and patients with MCI due to AD or mild to moderate AD.	NCT02754830	Eli Lilly and Company	Phase 1	Study was completed in 2023. 5% of frequency threshold of other adverse event (not serious) was reported including abdominal pain, diarrhea and vomiting.
TPI-287	• Microtubule stabilizers • To evaluate the highest intravenous dose of TPI-287 that is safe and tolerable for mild to moderate AD, measure its pharmacokinetics, and assess its preliminary efficacy on disease progression.	NCT01966666	University of California, San Francisco	Phase 1	Study was completed in 2019. In this randomized clinical trial, TPI-287 was less tolerated in patients with AD than in those with 4RT owing to the presence of anaphylactoid reactions. The ability to reveal different tau therapeutic effects in various tauopathy syndromes suggests that basket trials are a valuable approach to tau therapeutic early clinical development (Tsai et al., 2020).
AADvac1	• Active immunotherapy • To evaluates the safety and efficacy of AADvac1 in the treatment of patients with mild AD.	NCT02579252	Axon Neuroscience SE	Phase 2	Study was completed in 2019. The Phase 1 (2015) of AADvac1 had a favorable safety profile and excellent immunogenicity, however, the phase 2 trial outcomes were not mentioned.
BIIB092	• Passive immunotherapy • To assess BIIB092’s safety and tolerability in MCI owing to AD or mild AD. Secondary objectives of the placebo-controlled period include evaluating the efficacy of multiple doses of BIIB092 in slowing cognitive and functional impairment in participants with MCI due to AD or mild AD and its immunogenicity.	NCT03352557	Biogen	Phase 2	Terminated in 2022 based on lack of efficacy following the placebo-controlled period readout.
**DMTs employing other pathways**					
AGB101	• Neuroprotection • To determine whether AGB101 slows cognitive and functional impairment as measured by changes in the CDR-SB score compared to placebo in participants with MCI due to AD, also known as prodromal AD.	NCT03486938	AgeneBio	Phase 2 and 3	Study was completed in 2022. Three subjects were randomized and assigned to receive AGB101 but were not treated, lowering the total number of at-risk participants treated with AGB101 to 78. Further elaboration of the drugs intervention was not specified.
BHV4157	• Neuroprotection • To evaluate the efficacy and safety of BHV-4157 in patients with mild to moderate AD	NCT03605667	Biohaven Pharmaceuticals, Inc.	Phase 2	Study was completed in 2021. Eligible participants who completed the double-blind treatment phase had the opportunity to receive open-label troriluzole for up to 48 weeks in an open-label extension (OLE) phase.
Icosapent ethyl	• Neuroprotection • To assess whether icosapent ethyl beneficially affects intermediate physiological measures associated with onset of AD in order to evaluate whether larger, multi-site, longer-duration Alzheimer’s prevention trials are warranted to assess more definitive clinical outcomes.	NCT02719327	VA Office of Research and Development	Phase 2 and 3	Study was completed in 2023, however, the outcomes of this study were not specified.
ALZT-OP1a plus ALZT-OP1b	• Anti-inflammatory • To determine whether ALZT-OP1 combination treatment (ALZT-OP1a + ALZT-OP1b) will slow down, arrests, or reverse cognitive and functional decline, in subjects with evidence of early-stage AD.	NCT02547818	AZTherapies, Inc	Phase 3	Study was completed in 2020, however the outcomes of this study were not specified.
COR388	• Anti-inflammatory • To assess the efficacy, safety, and tolerability of 2 dose levels of COR388 in subjects with a clinical diagnosis of mild to moderate AD dementia.	NCT03823404	Cortexyme Inc.	Phase 2 and 3	Study was completed in 2023. 2.34% mortality was reported for COR388 80 mg BID, while 0.47% mortality was reported for COR388 40 mg BID. 11.68% serious adverse events were reported for COR388 80 mg BID, while 9.43% serious adverse events were reported for COR388 40 mg BID.
Masitinib	• Anti-inflammatory • To assess the safety and efficacy of masitinib for the treatment of mild to moderate AD.	NCT01872598	AB Science	Phase 3	Study was completed in 2020, however, the outcomes of this study were not specified.
GRF6019	• Anti-inflammatory • To evaluate the safety, tolerability, and feasibility of GRF6019, a plasma-derived product, administered as an intravenous (IV) infusion, to subjects with mild to moderate AD.	NCT03520998	Alkahest, Inc.	Phase 2	Study was completed in 2019. Results showed GRF6019 at high dose improved MMSE, ADASCog and ADCS-ADL. However, GRF6019 caused serious adverse event such as infusion related reaction and pulmonary embolism.
mAbs AL002 and AL003	• Anti-inflammatory • To systematically assess the safety (including immunogenicity) and tolerability, pharmacokinetics (PK), and pharmacodynamics (PD) of AL002.	NCT03635047	Alector Inc.	Phase 1	Study was completed in 2020, however, the outcomes of this study were not specified.
	• Anti-inflammatory • To systematically assess the safety (including immunogenicity) and tolerability, pharmacokinetics (PK), and pharmacodynamics (PD) of AL003	NCT03822208	Alector Inc.	Phase 1	Study was completed in 2021, however, the outcomes of this study were not specified.
A combination of losartan, amlodipine, atorvastatin, and exercise	• Metabolic effects • The rrAD study will determine effects of aerobic exercise training and intensive vascular risk reduction on cognitive performance in older adults who have high risk for AD.	NCT02913664	University of Texas Southwestern Medical Center	Phase 2 and 3	Study was completed in 2021, however, the outcomes of this study were not specified.
AstroStem	• Metabolic effects • To evaluate the safety and efficacy of AstroStem, autologous adipose tissue derived mesenchymal stem cells, in patients with AD.	NCT03117738	Nature Cell Co. Ltd.	Phase 1 and 2	Study was completed in 2021. There were 27.27% serious adverse events complicated with the AstroStem compared to placebo, including diarrhea, neoplasms, and pulmonary embolism.
	• Metabolic effects • To test the safety and efficacy of LMSCs (Longeveron Mesenchymal Stem Cells) for the treatment of subjects with clinically diagnosed AD.	NCT02600130	Longeveron Inc.	Phase 1	Study was completed in 2020, however, the outcomes of this study were not specified.

Aβ, amyloid beta; AD, Alzheimer’s disease; MCI, Mild Cognitive Impairment; rrAD, risk reduction for Alzheimer’s disease; CDR-SB, clinical dementia rating-sum of boxes; Im, intramuscular; ADAS-Cog, Alzheimer’s disease assessment scale-cognitive subscale.

## (i) Reduction of Aβ42 production

### γ-secretase inhibitors

As per the amyloid hypothesis, the amyloidogenic pathway is facilitated following the successive cleavage of APP by BACE1 and γ-secretase. Accordingly, the suppression of these enzymes has been regarded as a significant focus for therapeutic strategies. Unfortunately, with regards to γ-secretase, apart from APP, this specific enzyme interacts with numerous other substances and cleaves various transmembrane proteins. This fact likely accounts for the recent failures in clinical trials involving γ-secretase inhibitors. Semagacestat was linked to a deterioration in daily functioning and an increased incidence of infections and skin cancer (Doody et al., 2013). Avagacestat was associated with a higher rate of cognitive decline and adverse effects that limited the dosage, such as skin cancer (Coric et al., 2015). Tarenflurbil, on the other hand, exhibited poor ability to penetrate the brain (Muntimadugu et al., 2016). The presence of significant safety issues surrounding γ-secretase inhibitors renders γ-secretase an unsuitable target for treating AD (Penninkilampi et al., 2016). Thorough investigations on this crucial enzyme are necessary to enable the development of a safe therapeutic approach to target γ-secretase (Steiner et al., 2018). There are presently no γ-secretase modulators being investigated in phase 1–3 clinical studies (Huang et al., 2023).

### BACE inhibitors

Within the amyloidogenic route, β-secretase cleaves APP, resulting in the production of Aβ peptides, which ultimately leads to neurodegeneration (Das and Yan, 2017). Recently, several clinical trials have been conducted for BACE inhibitors. However, a significant number of these trials have been unsuccessful in demonstrating positive results in people with mild to moderate AD, despite using a rigorous research design that involved randomly assigning participants to either the treatment or placebo group.

The majority of BACE1 inhibitors, including LY2886721 [NCT01561430], Elenbecestat (E2609) [NCT02956486], CNP520 [NCT02565511], Verubecestat [NCT01953601], and Atabecestat [NCT02569398], have been discontinued from clinical trials (Das and Yan, 2017; Egan et al., 2019; Imbimbo and Watling, 2019).

### α-secretase modulators

APP undergoes processing by the α-secretase enzyme in the non-amyloidogenic route. α-secretase enzymatically breaks the peptide link between lysine 16 and leucine 17 in APP. This process generates two products: soluble amyloid precursor protein (sAPPα) and a membrane-bound fragment called C83. C83 is then subjected to additional processing by γ-secretase, resulting in the production of p3 and AICD (Folch et al., 2018). Thus, α-secretase reduces the production of Aβ and also demonstrates neuroprotective effects (Lichtenthaler and Haass, 2004). Therefore, α-secretase enhancers offer a compelling approach for the advancement of DMTs. Various substances have been examined to activate the non-amyloidogenic pathway. However, scientists are currently anticipating the development of a drug that can activate the non-amyloidogenic pathway in order to reduce the production of Aβ. The clinical trial stage is hindered by a lack of selectivity toward α-secretase and the presence of toxicities, resulting in a reduced number of compounds being reached.

Etazolate (EHT0202) functions as a selective modulator of GABA receptors and promotes the nonamyloidogenic α-secretase pathway. A prior phase 2 trial demonstrated that the drug was safe and well tolerated in patients with mild to moderate Alzheimer’s disease. Nevertheless, the advancement of etazolate in phase 3 trials has not continued (Vellas et al., 2011).

## (ii) Reduction of Aβ-plaque burden

### Aggregation inhibitors

Aggregation inhibitors directly interact with the Aβ peptide to prevent the development of Aβ42 fibers. As a result, they are seen as promising treatment agents for AD. Tramiprosate has undergone preclinical and clinical investigations to assess its effectiveness in treating AD (Caltagirone et al., 2012; Hey et al., 2018). Tramiprosate is an oral medication that inhibits the aggregation of amyloid proteins. It has been studied in patients with mild to moderate AD (Abushakra et al., 2016). Regrettably, tramiprosate proved unsuccessful in the phase 3 clinical study due to its adverse effects on the gastrointestinal system, including causing nausea and vomiting (Abushakra et al., 2016). Following the unsuccessful phase 3 clinical studies, tramiprosate was subsequently marketed as a dietary supplement. In addition, a prodrug called ALZ-801, derived from tramiprosate, exhibits a unique ability to counteract amyloid oligomers. ALZ-801 is currently in phase-2 clinical trial at the moment the article is written (clinicaltrials.gov). It has been speculated that ALZ-801 be granted fast-track designation by the US FDA for the treatment of AD (Gupta and Samant, 2021).

## (iii) Enhancing Aβ clearance (active or passive immunotherapy)

The two primary immunotherapeutic strategies now being investigated in clinical and preclinical trials to enhance the removal of Aβ are active and passive immunization. Active immunization involves the activation of T and B cells, which in turn stimulates the phagocytic capacity of microglia, resulting in an immunological response. Passive immunization primarily focuses on stimulating the immune response against Aβ through the use of monoclonal or polyclonal antibodies (Gupta and Samant, 2021).

### Active Aβ immunotherapy

The fundamental advantage of active immunotherapy is that it stimulates the creation of endogenous antibodies without the need for repeated administration. However, no significant therapeutic benefit has been documented in AD patients, and due to the possibility of unpredictable immune response with potentially severe adverse effects, no vaccine has yet been approved for commercialization (Kwan et al., 2020). CAD106 is a proactive Aβ immunotherapeutic drug that underwent phase 2 and 3 clinical trials to assess its potential in delaying the onset and advancement of clinical symptoms related to AD in individuals who are at risk of developing such symptoms based on their age and genotype. Nevertheless, CAD106 was discontinued as a result of unforeseen alterations in cognitive performance, reduction in brain capacity, and decreased in body weight (clinicaltrials.gov).

The efficacy of ABvac40 was assessed in a phase 2 clinical trial, making it the initial active immunization targeting the C-terminal region of Aβ40. A phase 1 clinical trial was undertaken including patients diagnosed with mild to moderate AD, ranging in age from 50 to 85 years. No signs of vasogenic oedema or microhaemorrhages were found. Anti-Aβ40 antibodies were specifically produced in 92% of those who had ABvac40 injections (Lopez et al., 2019). A phase 2, double-blind, parallel-group, placebo-controlled and 6-month randomized clinical trial to evaluate the efficacy and safety of GV1001 in Alzheimer patients was completed in 2019. The findings demonstrate that 1.12 mg of GV1001 successfully achieved the primary objective of a statistically significant distinction. GV1001 demonstrated excellent tolerability without any safety issues (Koh et al., 2021). The efficacy of ACC-001 (vanutide cridificar), a vaccine targeting Aβ, was evaluated in phase 2a extension trials involving individuals diagnosed with mild to moderate AD. The administration included the use of QS-21 adjuvant. Extended treatment with this combination was highly well-tolerated and resulted in the most elevated levels of anti-Aβ IgG antibodies in comparison to alternative therapy options (Hull et al., 2017).

### Passive Aβ immunotherapy

The use of monoclonal antibodies is limited by the development of dose-dependent side effects, which can be seen in one-third of individuals with “amyloid-related imaging abnormalities” (ARIAs) (Piazza and Winblad, 2016). ARIAs can lead to the onset of vasogenic edema (ARIA-E) or cerebral micro-hemorrhages (ARIA-H), which are distinguished by neuroimaging evidence of hemosiderin deposits. ARIAs were identified in clinical trials assessing the safety and efficacy of practically all monoclonal antibodies, and were generally dose dependent (Bateman et al., 2023).

Aducanumab, also known as Aduhelm, is a monoclonal antibody of the immunoglobulin gamma 1 (IgG1) class that has a strong attraction to and binds to the N-terminus of Aβ fibrils, preventing the aggregation of amyloid proteins (Arndt et al., 2018). The initiation of two phase 3 clinical trials, ENGAGE and EMERGE investigations, began in August 2015. Aducanumab (BIIB037) has demonstrated substantial improvements in cognitive and functional domains, including memory, orientation, and language. Aducanumab (BIIB037) consistently and convincingly decreased the quantity of amyloid plaques in the brain. In June 2021, the FDA granted immediate approval for Aduhelm (aducanumab-avwa) to treat AD based on its observed effects. It was declared as a newly authorized drug for people with Alzheimer’s. Following approval, pharmaceutical companies are required to conduct Phase IV confirmatory trials to assess the efficacy of their medicines. If the drug fails to perform as expected, the FDA has the authority to withdraw it from the market. The controversial approval of Aducanumab, its disputed clinical impact, and subsequent decline all contribute to the anti-amyloid therapy debate. While Aβ accumulation is important for AD pathogenesis, it does not appear to be sufficient to trigger neurodegenerative alterations and cognitive impairment. Future clinical trials should not overlook the critical connection between amyloid, tau, and neuroinflammation to raise the likelihood of clinical efficacy (Golde, 2023).

Lecanemab, also known as Leqembi, is a humanized IgG1 antibody that is generated from mAb158. It specifically attaches to soluble Aβ protofibrils (Tucker et al., 2015). The US FDA granted permission on 6 January 2023, via an expedited approval process due to the presence of evidence indicating amyloid elimination in a phase 2 trial (NCT01767311) and the potential for clinical advantages (Canady, 2023). An 856-patient double-blind, placebo-controlled phase 2 trial was conducted to study individuals with AD who had either MCI or mild dementia. The participants were confirmed to have amyloid pathology using amyloid PET or CSF Aβ1-42 testing. The findings demonstrated a notable and dosage-dependent decrease in amyloid plaques in the lecanemab group (10 mg/kg, administered through intravenous infusion every 2 weeks) from the initial measurement to week 79, in comparison to the placebo group. Currently, there are three ongoing phase 3 clinical trials for lecanemab (clinicaltrials.gov).

Donanemab is a monoclonal antibody that has been humanized from the mouse antibody mE8-IgG2a. It identifies Aβ (3–42), a clustered version of Aβ discovered in amyloid plaques (Irizarry et al., 2016). Upon examination of postmortem brain samples from people with AD or Down syndrome, it was shown that the substance was attached to almost one-third of amyloid plaques. Furthermore, it exhibited a robust reaction with the central part of the plaque (Bouter et al., 2022). Phase II TRAILBLAZER-ALZ research assessed the safety, tolerability, and effectiveness of donanemab, both as a standalone treatment and in conjunction with the Beta-Secretase 1 (BACE1) inhibitor LY3202626, which was produced by Eli Lilly and Company. The study spanned a duration of 18 months. The experiment successfully achieved its primary objective of significantly postponing the deterioration, as measured by iADRS scores, by 32% compared to the placebo. The decrease in amyloid accumulation was found to be associated solely with an improvement in iADRS scores in individuals who carry the ApoE4 gene (Shcherbinin et al., 2022). Donanemab effectively decreased the accumulation of tau in the temporal, parietal, and frontal lobes, and resulted in a significant 24% reduction in plasma pTau217 levels in the treatment group. In contrast, the placebo group had a 6% increase in plasma pTau217 levels by the end of the study (Pontecorvo et al., 2022). TRAILBLAZER-ALZ 2 Phase 3 study (donanemab-azbt, 350 mg/20 mL once-monthly injection for IV infusion) has been approved recently (2 July 2024) by FDA (Wall, 2024). This drug will become a treatment option for adults with early symptomatic Alzheimer’s disease, including people with mild cognitive impairment (MCI) and mild dementia with confirmed amyloid plaques. Lilly is currently conducting several clinical trials with donanemab. These trials include TRAILBLAZER-ALZ 3 (currently recruiting), which aims to prevent symptomatic Alzheimer’s disease in participants with preclinical AD; TRAILBLAZER-ALZ 5 (currently recruiting), a registration trial for early symptomatic AD that is currently recruiting in China and Korea; and TRAILBLAZER-ALZ 6 (currently recruiting), which focuses on advancing our understanding of ARIA through novel MRI sequences, blood-based biomarkers, and various donanemab dosage regimens (Wall, 2024). The TRAILBLAZER-ALZ 4 clinical study, which investigated the efficacy of donanemab compared to aducanumab in clearing brain amyloid plaques in individuals with early symptomatic AD, completed in 2023 (clinicaltrials.gov).

Solanezumab, a humanized monoclonal antibody, targeting the mid-domain of the Aβ peptide to enhance Aβ clearance (Honig et al., 2018). The Phase III clinical trial of solanezumab (LY2062430) ended in October 2019 [NCT02760602] due to the failure of the EXPEDITION 3 study. Other Phase III studies with the same substance, solanezumab [NCT01900665; NCT01127633], also failed as a result of the EXPEDITION 3 study. Despite the disappointing outcomes of these studies, solanezumab is still being tested in patients with a genetic mutation that may put them at risk of developing AD in a Phase 2/3 clinical trial called DIAN-TU [NCT01760005] (clinicaltrials.gov). ALZ-801 is a pharmacologically inactive derivative of tramiprosate, a tiny molecule that can counteract Aβ oligomers and prevent their aggregation (Hey et al., 2018). The APOLLOE4 (NCT04770220) phase 3 trial is assessing the safety and effectiveness of ALZ-801 in patients with early AD who have two copies of the ε4 allele on the apolipoprotein E gene (APOE4/4). A separate phase 2 clinical trial (NCT04693520) is currently examining the impact of oral ALZ-801 on individuals with early AD who possess the APOE4/4 or APOE3/4 genotype and have biomarkers indicating the presence of core AD pathology. The trial is evaluating the effectiveness, safety, and capacity to be tolerated of ALZ-801. ABBV-916 is a monoclonal antibody that targets Aβ. It identifies N-terminal truncated Aβ that has been changed with pyroglutamate at position 3 (N3), which is a variant of Aβ that forms aggregated amyloid plaques. The clinical trial for ABBV-916, consisting of two phases, is now in progress (NCT05291234) (clinicaltrials.gov).

## Tau-related DMT strategies

The failure of multiple Phase II/III trials in AD that focused on reducing Aβ accumulation has led to a growing interest in alternate treatments for tau pathology (Panza et al., 2016). Tau proteins, often referred to as axonal microtubule-associated protein (MAP), play a crucial role in controlling the assembly and arrangement of microtubules, as well as the transportation of organelles within axons. Excessive tau phosphorylation has been proposed as a possible factor in the development of neurofibrillary tangles in AD (Götz et al., 2012). In individuals with AD, the process of hyperphosphorylation of tau proteins leads to the separation of tau proteins from the microtubules. This disruption of the axonal transport structure results in a lack of nutrients reaching the neurons, ultimately leading to their death (Terwel et al., 2002).

### Tau phosphorylation and aggregation inhibition

Tau phosphorylation and aggregation inhibitors are employed to mitigate tauopathy and hinder tau aggregation. TRx0237 is a second-generation inhibitor of tau protein aggregation that underwent Phase III clinical trials to assess the safety and effectiveness of TRx0237 at doses of 16 mg/day and 8 mg/day in the treatment of individuals with AD. The trial was completed in May 2023 [NCT03446001]. LY3303560 is another compound that acts as a tau phosphorylation inhibitor. It completed its Phase II clinical trial in October 2023. GSK3 inhibitors are utilized as a means to decrease tau hyperphosphorylation, which is primarily caused by the enzyme responsible for turning tau into hyperphosphorylated tau protein (Hooper et al., 2008). Tideglusib, also known as NCT00948259, is a GSK3 inhibitor. It is a small-molecule medicine that can be taken orally and is designed to decrease the excessive phosphorylation of tau protein. Noscira SA is the company responsible for developing this therapy. Tideglusib commenced Phase II clinical trials and was administered to individuals with mild to moderate AD in December 2008. Nevertheless, tideglusib was determined to be safer in the trial. However, it did not meet its primary endpoint, and as a result, some of the secondary endpoints did not demonstrate any meaningful therapeutic advantages (Serenó et al., 2009).

Various techniques have been employed to lower the amounts of various forms of tau protein (including monomers, oligomers, filaments, granules, fibrils, and insoluble aggregates) in AD. Considering tau aggregation inhibitors as a primary focus could be beneficial for managing AD (Bulic et al., 2013). Tau-tau interactions play a crucial role in the development of neurofibrillary tangles (NFTs). The Phase III clinical trial evaluated the efficacy of low dose, 4 mg twice a day, Leuco-Methylthioninium Bis (Hydroxymethanesulfonate) monotherapy in treating mild AD patients. The modified primary outcome measure used in this trial was cohort analysis, which yielded favorable outcomes (Wilcock et al., 2018).

### Microtubule stabilizers

Tau hyperphosphorylation in AD is linked to the disruption of microtubules. AD treatment has been the subject of preclinical and clinical experiments with various microtubule stabilizers (Brunden et al., 2011). Paclitaxel, an anti-mitotic drug, was discontinued from the trial because of its limited ability to pass through the BBB (Fellner et al., 2002; Zhang et al., 2005; Zempel et al., 2010). The recruitment of individuals for the Phase I trial of TPI287, a synthetic epothilone derivative, focusing on safety, tolerability, pharmacokinetics, and pharmacodynamics, was completed in April 2020 (Brunden et al., 2010).

### Anti-tau immunotherapy

Recent evidence from multiple animal models indicates that focusing on p-tau epitopes is a viable strategy to stimulate antibody responses that can facilitate the removal of tau (Wischik et al., 2015). Therefore, several immunotherapy efforts, both active and passive, have progressed to clinical trials for the treatment of AD (Medina, 2018).

### Active immunotherapy

AADvac1, which incorporates a synthetic tau peptide, underwent a phase 2 clinical trial for those with mild to severe AD. The clinical trial was completed in November 2019 (NCT02579252) (Wischik et al., 2015).

### Passive immunotherapy

ABBV-8E12, a humanized anti-tau monoclonal antibody, was evaluated in a phase 2 clinical trial including patients with early AD (NCT02880956) (Budur et al., 2017). BIIB092 is a monoclonal antibody that has been humanized to target tau fragments. These fragments are obtained from the stem cells of a patient with familial AD (Wilcock et al., 2018). A phase 2 clinical trial evaluates the safety and effectiveness of the drug in individuals with amnestic moderate cognitive impairment (AD MCI) and mild AD (Cummings et al., 2022).

## DMTs employing other pathways

### Neuroprotection

These group of drugs refers to preservation of neural tissue from damage or degeneration. AGB101, a low-dose extended-release form of levetiracetam, is a modulator of SV2A. It completed a phase 3 clinical trial in September 2023 as a repurposed medication. Originally approved for use in a different indication, namely MCI owing to AD, rather than epilepsy. The purpose is to decrease excessive neural activity caused by Aβ (NCT03486938) (clinicaltrials.gov).

BHV4157, also known as troriluzole, is a substance that modulates glutamate and decreases the amounts of glutamate in synapses. It has undergone a phase 2 clinical trial (NCT03605667) and the trial was finished in December 2023. The clinical trial aimed to test the efficacy and safety of BHV-4157 in patients diagnosed with mild to moderate AD (clinicaltrials.gov).

Icosapent ethyl is a refined version of eicosapentaenoic acid (EPA), which is an omega-3 fatty acid. The purpose of the phase 3 clinical trial (NCT02719327) was to determine whether icosapent ethyl, a medication, can protect neurons from disease pathology and positively impact intermediate physiological measures that are associated with the onset of AD. The trial aimed to evaluate whether larger, multi-site, longer-duration trials are necessary to assess more definitive clinical outcomes related to Alzheimer’s prevention (clinicaltrials.gov).

### Anti-inflammatory effects

Neuroinflammation has been implied as a potential cause of AD for over 30 years. However, only recently the research into neuroinflammation gained momentum, likely due to two significant findings. Firstly, there is evidence indicating that activated glial cells play a role in the development of brain lesions in AD. Secondly, epidemiological studies have shown that patients with rheumatoid arthritis, who have been treated with anti-inflammatory drugs for many years, are protected from developing AD (McGeer et al., 2016).

These are the anti-inflammatory drugs that have undergone completion in clinical trials:

ALZT-OP1a plus ALZT-OP1b is a combination of cromolyn, which is a mast cell stabilizer, and ibuprofen, which is an anti-inflammatory drug. The purpose of the phase 3 clinical trial (NCT02547818) was to analyze the safety and tolerability of the combination medication ALZT-OP1, as well as its effectiveness in slowing down, arresting, or reversing cognitive and functional deterioration in individuals with early-stage AD. The experiment also aimed to measure efficacy using the CDR-SB scale.

COR388 is a substance that specifically targets a type of bacteria that causes periodontal disease. The efficacy, safety, and tolerability of two dose levels of COR388 were evaluated in a Phase 2/3 clinical trial (NCT03823404). The study was conducted in a randomized, double-blind, placebo-controlled manner and included participants having a clinical diagnosis of mild to severe AD dementia.

Masitinib functions as a specific tyrosine kinase inhibitor and a regulator of neuroinflammation by targeting mast cells. The drug’s safety and efficacy in treating mild to moderate AD were evaluated in a phase 3 clinical trial (NCT01872598). The drug masitinib was given as an additional treatment to patients who had already been receiving treatment with a consistent dose of cholinesterase inhibitor (donepezil, rivastigmine, or galantamine) and/or memantine for at least 6 months.

Elderberry Juice enhances mitochondrial function by acting as a potent antioxidant, thanks to its high content of anthocyanins (NCT02414607). GRF6019, a fraction of human plasma protein, is administered through infusions with the aim of counteracting brain neuroinflammation through young blood parabiosis (NCT03520998, NCT03765762). These agents have successfully passed the phase 2 clinical trials (Cummings et al., 2019).

In phase 1, anti-inflammatory drugs investigated included mAbs AL002 and AL003 (NCT03635047, NCT03822208) (Cummings et al., 2019).

### Metabolic effects

Utilizing a combination of losartan, amlodipine, atorvastatin, and exercise is a recommended treatment strategy for repurposing, aiming to significantly decrease vascular risk and preserve cognitive function. The assessment was carried out in a phase 3 clinical trial (NCT02913664) to ascertain the impact of aerobic exercise training and intense vascular risk reduction on cognitive performance in older persons who are at a high risk for AD (Cummings et al., 2019).

### Stem-cell approaches

AstroStem is a therapeutic procedure that utilizes stem cells obtained from a person’s adipose tissue. The treatment involves intravenous administration of these stem cells, which is repeated 10 times. AstroStem was evaluated in a phase 1/2 clinical trial (NCT03117738), while the treatment including human mesenchymal stem cells (hMSCs) was evaluated in a phase 1 clinical trial (NCT02600130) (Cummings et al., 2019).

## Phytochemical approaches

Ongoing research efforts are actively exploring the potential protective benefits of plant phytochemicals as nutraceutical agents against neuropathological conditions associated with AD. This strategy holds significant promise due to their therapeutic potential, minimal side effects, diverse molecular targets, potential for disease modification, dietary feasibility, and demonstrated neuroprotective effects in preclinical studies (Abdul Manap et al., 2019a; Ayaz et al., 2019; Rahman et al., 2021). Further research in this area may lead to the development of novel preventive and therapeutic strategies for AD. The summary of various phytochemicals undertaken by previous studies is demonstrated in the Table 5 below.

**TABLE 5:** An overview of the several phytochemicals employed in earlier research as neuroprotective agents against AD.

Phytochemicals	Intervention	Study setting	Outcome summary	References
Curcumin	Investigation on curcumin and piperine against Aβ-induced neurotoxicity in cell line AD model	*In vitro* model	Combination of curcumin and piperine protected SH-SY5Y cells against Aβ-induced cytotoxicity, fibrillation, and oxidative damage.	Abdul Manap et al., 2019b
Resveratrol (RES)	Investigation on the impact of RES, both independently and in conjunction with vitamin E on rats afflicted with AD induced by scopolamine (SCO).	*In vivo*	Following RES treatment, alterations induced by SCO in AChE, protein carbonyl, and TNF-α resulted in elevated antioxidant levels, mitigated SCO-induced lipid peroxidation, and reversed SCO-mediated changes, outperforming the drug donepezil.	Foudah et al., 2023
Epigallocatechin Gallate (EGCG)	EGCG is evaluated as a small molecule capable of disaggregating tau amyloid fibrils.	*In vitro*	EGCG molecule structure has shown to stack in polar clefts between the pathologically defined paired helical protofilaments in AD.	Seidler et al., 2022
Ginkgo Biloba Extract	To describe the study that uses EGB761 as a dual target for AD.	*In silico* docking analysis	According to molecular docking and network pharmacology study, the highly active phytocompounds of EGB761, particularly quercetin, kaempferol, and isorhamnetin, exhibited more robust activity against AChE and GSK3 than the reported synthesized medication.	Singh et al., 2023
Quercetin	To evaluate quercetin’s neuroprotective impact on hallmark genes in rats with AlCl3-induced AD. AlCl3 group, and 60 days of co-administration with AlCl3 + Q50.	*In vivo* animal model (Wistar male rats)	It reduced APP expression, while increasing ADAM 17 expression in the non-amyloidosis pathway.	Elreedy et al., 2023
Polyphenols	Investigation on the various dietary polyphenols such as rosmarinic acid, ellagic acid, and cinnamic aldehyde as neuroprotective and pro-cognitive agents via various molecular mechanisms.	*In vivo* model	These natural compounds have been shown to have a number of neuroprotective and cognition-enhancing effects due to their anti-amyloidogenic and anti-aggregate activity.	Caruso et al., 2022
Ginsenosides (Rg1)	Investigation on Ginsenoside Rg1 (Rg1) as a neuroprotective agent against animals with memory impairment.	*In vivo*	Through the regulation of the Wnt/GSK-3β/β-catenin signaling pathway, it has been observed that Rg1 administered at moderate and high doses exhibits the potential to mitigate oxidative stress-induced damage, ameliorate neuroinflammation, safeguard neurons, and ultimately enhance cognitive impairment in the AD model of the tree shrew.	Yang et al., 2022
Crocus sativus	To investigate the anti-inflammatory and anti- Aβ aggregation properties of saffron	*In vivo*	The animal studies have provided evidence for the anti-inflammatory and anti- Aβ aggregation properties of saffron.	Marzabadi et al., 2022
Hesperidin	To explore the potential neuroprotective attributes of hesperidin and naringin via an AD model in SK-N-AS cells that was employing Aβ25-35.	*In vitro*	In the AD model cells, the intensity of Aβ was notably diminished upon treatment with both hesperidin and naringin. Additionally, both flavonoids exhibited a significant reduction in the intensity of α-synuclein within the SK-N-AS cells and AD model cells.	Kuşi et al., 2023
Lycopene	To investigate the neuroprotective properties of lycopene and the underlying mechanisms involved, employing a murine model in which Aβ1–42 was administered intracerebroventricularly (ICV).	*In vivo*	Notably, the supplementation of Lycopene Micelles in Olive Oil (LME) resulted in a remarkable mitigation of astrocytosis and microgliosis, a reduction in malondialdehyde production, and a restoration of antioxidant capacities.	Guo et al., 2023
Olea europaea (Oleuropein)	Investigation on olive leaf (OL), as well as its compounds Oleuropein (OLE) and Hydroxytyrosol (HT), as a dual capacity for diminishing of the formation of Aβ and neurofibrillary tangles	*In vitro*	The efficacy of OL and the bioactive compounds within this by-product of the olive tree has been demonstrated in mitigating, and potentially preventing, various processes associated with AD.	Romero-Márquez et al., 2023

## Non-pharmacological approaches

### Cognitive training

Memory difficulties are a distinguishing hallmark of the early stages of AD and vascular dementia (Karantzoulis and Galvin, 2011). Interventions that target these cognitive deficiencies and the concomitant difficulty with daily activities are gaining popularity. Cognitive training and cognitive rehabilitation are non-pharmacological interventions used to improve cognitive and non-cognitive outcomes (Irazoki et al., 2020). Interventions that directly or indirectly target cognitive functioning are distinguished from those that primarily target behavioral (for example, roaming), emotional (for example, anxiety), or physical (for example, sedentary lifestyle) function (Mandolesi et al., 2018). There are various forms of cognition-based therapies that have been described that are focusing on reasoning, speed of processing information and memory (Harvey, 2019). The brain therapy and exercises can be as simple as doing thing with the non-dominant hands. This activity requires little to no cost and can be done anywhere anytime of the day which make it as an easy approach to the patient. New and challenging activity will stimulate the brain more rather than doing same thing every day (Alzheimer’s & Dementia Resource Center, 2023). Hence, learning new language at the old age is very recommended as a form of exercises that will help to decrease the rate of brain declination. If the person is a person who likes to have fun and laidback, playing boardgames and card games within the Alzheimer’ community or together with their family members are highly recommended. Activities like this will not only help delaying the brain declines but making social connection with other people despite suffering from the disease (Dementia and Alzheimer’s, 2022). Based on the research conducted by Trebbastoni et al. (2017), the data for the usefulness of cognitive training in AD is still weak. This study demonstrates that a six-month intensive cognitive training program may aid in the preservation of cognition in people with mild-to-moderate AD. Indeed, post-intervention and six-month follow-up outcome tests show that this intervention is effective in improving numerous cognitive processes, including memory (Trebbastoni et al., 2017). To corroborate findings, future randomized clinical trials should be designed as multicentre research trials with larger patient samples and longer intervention and post-intervention observation periods (Nair, 2019). Furthermore, because cognitive training has no side effects, it is clearly preferred in circumstances where drug-drug interactions, drug-related side effects, or contraindications exclude a pharmaceutical therapy to the disease (Giuli et al., 2016).

### Physical exercise, ergotherapy and brain simulation

Physical exercise always been known for its benefit for human overall health. Alzheimer patient might also benefit from doing physical exercise (Better Health Channel, 2023). According to a University of Wisconsin study, those over 60 who were at high risk of AD and who engaged in moderate exercise for 30 min five days a week experienced less memory and cognitive issues as well as a decreased chance of getting the condition (Johnson et al., 2018). Study from the University of Kansas discovered that some participants with Alzheimer’s were able to improve their memory test scores and even increase the size of their brain’s hippocampus, an area of the brain important for learning and memory that is typically impacted early in the AD process, after routinely exercising (Morris et al., 2017). To recently, exercise studies have either been too small-scale to be conclusive or have yielded mixed results in terms of their impact on memory and brain function (Loprinzi et al., 2023). It is believed that the most beneficial aerobic exercise for the brain is low intensity aerobic activity, such as brisk walking or swimming. Though the precise mechanism of exercise’s benefits is unknown, many hypothesize that it stems from enhanced blood vessel health and an increase in oxygen-rich blood flow to the brain, both of which enhance brain function (A Mental Workout, 2023). Ergotherapy is an occupational therapy to help people with dementia improve their self-care, productivity, and leisure/rest (Korczak et al., 2013). This allows dementia patients to improve their functional abilities in activities of daily living, social participation, quality of life, and life happiness (Ruthirakuhan et al., 2012).

The efficacy of current symptomatic medications such as cholinesterase inhibitors and memantine for the treatment of AD is limited to delaying the progression of symptoms (Hogan, 2014). However, some studies suggest that combining behavioral method and pharmacological treatment may optimize benefit for patient and caregiver, underlying the importance to develop nonpharmacological intervention programs (Magierski et al., 2020). Physical therapy assists dementia patients with mental health issues such as anxiety and depression (Orgeta et al., 2014). Regular exercise improves mood, reduces medication requirements, and aids the patient in controlling emotional symptoms of dementia such as restlessness, anger, and hostility. Physical therapy can provide major social benefits to dementia patients in addition to physical, cognitive, and emotional benefits (Jia et al., 2019). It lessens social anxiety, promotes stronger social relationships, and aids dementia patients’ efforts to keep their independence for as long as possible (Medical News Today, 2023).

## Emerging treatments

### Microbiota-gut-brain axis

Emerging studies has shed light on how gut bacteria and astrocytes communicate in both health and illness. Astrocytes are the most common glial cells in the CNS, and their array of functions is expanding, making them an increasingly popular research topic. Astrocytes play an important role in maintaining CNS homeostasis, and any disturbances in their activity contribute to the development of neurological disorders. Importantly, emerging investigations have revealed that bidirectional signaling between astrocytes and microglia drives neuroinflammation and neurodegeneration (Lee et al., 2022; Patani et al., 2023).

The effects of gut microbiota alteration on astrocytes in AD were very recently discovered. Perturbation of the gut microbiota has also been demonstrated to minimize reactive astrogliosis, promote astrocyte homeostasis, and protect against amyloidosis and tau-mediated neurodegeneration. Interestingly, these effects appear to be more prevalent in male mice (Chandra et al., 2023; Seo et al., 2023).

Astrocytic responses to perturbation of the gut microbiota exhibit sexual dimorphism similar to that observed in microglia, highlighting the significance of incorporating gender effects into consideration in future studies. However, after microbiota restoration and SCFA supplementation, the neuroprotective effects of gut microbiota reduction were lessened. Specifically, in antibiotic-treated APP/PS1-21 mice, FMT from age-matched control mice recovered astrogliosis, but in GF TE4 mice, supplementation with SCFAs restored gliosis and tau pathology (Chandra et al., 2023; Seo et al., 2023). These animal studies, however, preliminary, showed that the gut microbiota plays a role in promoting the onset and advancement of AD pathology, including the regulation of astrocytic responses.

## 40 Hz gamma frequency brain rhythms

Tactile stimulation adds to the body of evidence demonstrating that non-invasive sensory stimulation of 40 Hz gamma frequency brain rhythms can mitigate AD pathology and symptoms. This effect has previously been demonstrated with light and sound by several research groups in both humans and animals (Peña-Ortega, 2019; Chan et al., 2021; Traikapi and Konstantinou, 2021). According to a recent study by MIT researchers, compared to untreated controls, Alzheimer’s model mice exposed to 40 Hz vibration for an hour a day for several weeks displayed better brain and motor performance (Suk et al., 2023).

The MIT group is not the first to demonstrate that gamma frequency tactile stimulation can influence brain activity and enhance motor function; however, they are the first to demonstrate that the stimulation can also prevent neurons from dying or losing their synapse circuit connections, lessen neural DNA damage, and lower levels of phosphorylated tau, a protein that is characteristic of AD. A team led by Tsai’s lab has shown in a number of papers that light flickering and/or sound clicking at 40 Hz (a technique known as GENUS for Gamma Entrainment Using Sensory stimuli) can lower tau and amyloid-beta protein levels, preserve synapses and prevent neuron death, and even maintain learning and memory in a range of AD mouse models. The team’s most recent pilot clinical trials revealed that 40 Hz light and sound stimulation was safe, effectively boosted brain connection and activity, and looked to have a major positive clinical impact on a small group of human volunteers who were suffering from early-stage AD (Suk et al., 2023).

In two widely used mouse models of Alzheimer’s neurodegeneration—the Tau P301S mouse, which mimics the disease’s tau pathology, and the CK-p25 mouse, which mimics the synapse loss and DNA damage seen in human disease—the new study examined whether whole-body 40 Hz tactile stimulation produced significant benefits. The primary motor cortex (MOp), where the brain generates movement commands for the body, and the primary somatosensory cortex (SSp), which processes touch sensations, were the focus of the team’s investigations.

The researchers vibrated mouse cages by placing them above speakers producing 40 Hz sound, which caused the cages to vibrate. The 40 Hz sound was played for all of the non-stimulated control mice since their cages were dispersed across the same space. Therefore, the addition of tactile stimulation was what caused the disparities between the stimulated and control mice to be measured. First, the scientists established that the 40 Hz vibration altered the neuronal activity in the brains of mice that were healthy—that is, animals without AD. Activity increased two-fold in the SSp and more than three-fold in the MOp, with a statistically significant increase in the latter case, as determined by the expression of the c-fos protein.

After learning that tactile stimulation at 40 Hz might raise brain activity, the researchers examined the effect on disease in the two mice models. The group employed female CK-p25 mice and male P301S mice to guarantee that both sexes were represented. When compared to unstimulated controls, P301S mice that were stimulated for three weeks demonstrated a notable preservation of neurons in both brain regions. Additionally, tau in the SSp by two measurements was significantly reduced in stimulated mice, and they also had comparable patterns in the MOp.

For six weeks, CK-p25 mice were subjected to vibration stimulation. In comparison to non-vibrated control mice, these mice exhibited increased levels of synaptic protein markers in both brain regions. Additionally, they displayed lower amounts of DNA damage. Lastly, the group evaluated the mice’s motor skills after exposing them to vibration vs. not. It was discovered that both mouse models could remain on a rotating rod for far longer. Additionally, P301S mice held onto a wire mesh for noticeably longer than control mice, but CK-p25 animals displayed a trend that was favorable but not statistically significant (Suk et al., 2023).

## Strategies to optimizing management for patient with AD

AD causes brain cells to die, causing the brain to function less effectively over time. This alters how a person behaves (National Institute on Aging, 2023). Behavioral symptoms can be one of the initial signs of dementing diseases, occurring before cognitive changes (Manni et al., 2023). These symptoms can occur at any point during the progression of the illness (Leocadi et al., 2023; Manni et al., 2023) and can vary depending on the severity of the dementia (Artaso-Irigoyen et al., 2004). Behavioral and psychological symptoms of dementia (BPSD) refer to non-cognitive symptoms that are frequently observed in individuals with AD (Fernández et al., 2010). Timely identification of BPSD is highly crucial, as these symptoms not only cause significant impairment in individuals with dementia, but also contribute to heightened stress for caregivers (Teixeira et al., 2024). Indeed, BPSD exacerbate difficulties in doing everyday tasks (Bélanger-Dibblee et al., 2023), expedite the deterioration of cognitive function (Honjo et al., 2020), and deteriorate the overall quality of life for patients (Kim et al., 2021). In addition, behavioral disorders are the primary cause of and result in the early institutionalization of patients (Gimeno et al., 2021), hence escalating the overall financial burden (Boafo et al., 2023). Nevertheless, once accurately diagnosed, many illnesses can be effectively managed with pharmaceutical interventions (López-Pousa et al., 2008), hence postponing the need for nursing home placement and enhancing the quality of life for both patients and caregivers.

A collaborative approach to tackling these complicated AD challenges is both realistic and effective. The team may also comprise professionals with knowledge of neurology, geriatric psychology, social work, clinical psychology, and elder law in addition to nurses and physician assistants (Physiopedia, 2023). The neurologist provides support in managing later stage neurologic signs of AD, including seizures, as well as in the differential diagnosis of patients demonstrating with atypical dementia presentation (Neugroschl and Wang, 2011). In addition to helping with the identification and psychopharmacologic treatment of behavioral issues like anxiety, psychosis, and depression, geriatric psychiatrists also aid in the differential diagnosis of difficult cases (Targum, 2001). The social worker may offer psychotherapy to patients and caregivers in addition to helping to preserve the stability of the patient’s family and finding and utilizing community resources for care (Ong et al., 2021). Expertise in behavioral responses to disorders like depression is provided by the clinical psychologist, who also helps with the identification of early-stage or dubious dementia. The elder law attorney can help with matters including guardianship and health-care financial planning (JSTOR, 2023). Other disciplines, including as pharmacy, nutrition, physical therapy, and occupational therapy, can also contribute significantly to management. Referral to a geneticist or a genetic counselor for the entire family and the patient is recommended for patients with early-onset familial AD (Grossberg and Desai, 2003).

## Conclusion and future perspectives

We represent the most sophisticated and extensive reviews on current diagnosis biomarkers and therapeutic approach achieved thus so far. It is now acknowledged that pathological alterations commence several years before the onset of clinical symptoms in diseases, and AD encompasses a range from individuals who show no clinical signs to those who are seriously impaired. Defining AD only based on its clinical symptoms is considered artificial. Therefore, attempts have been made to identify the disease by considering both clinical manifestations and biomarker evidence. The development of biomarkers has led to a change in how the disease is seen as a clinical and physiological entity. There is now a growing recognition that AD should not be seen as having distinct and well-defined stages, but rather as a complex process that progresses along a continuous continuum. Recognizing this concept is crucial for comprehending the progression of disease-modifying treatments and for implementing efficient diagnostic and illness management alternatives. The ATN classification, which focuses on biomarkers of AD, has evolved due to several factors. One key aspect is the recognition that the capacity to assess the fundamental biological elements of AD is significantly superior to that of other neurodegenerative disorders. Nevertheless, if and when biomarkers for new proteinopathies emerge, they could be incorporated. In the future, the inclusion of synaptic dysfunction as a category could be beneficial. This category could encompass many techniques such as FDG-PET, task-free functional MRI, EEG, MEG (magnetoencephalography), and the measurement of synapse-specific proteins in CSF. Nevertheless, if we define neurodegeneration as a gradual deterioration and constriction of neurons and their processes, accompanied by a commensurate decline in neuronal function, then synaptic dysfunction falls within the umbrella of neurodegeneration. In the future, it will be crucial to investigate novel biomarkers that extend beyond the amyloid and tau pathologies, as well as the longitudinal evolution of these biomarkers throughout the course of AD.

Apart from that, our review also primarily examines the present state of therapies in clinical trials and offers insight into the potential and promising targets for the development of drugs for AD. Currently, no cure or treatment can affect the progression of AD. However, drugs approved by the FDA can only provide relief from the symptoms in those with the condition. A multitude of drug candidates progressed through several stages of clinical trials, nevertheless, owing to unfavorable effects and insufficient therapeutic effectiveness, the majority of these substances failed to achieve success in Phase II/III trials. Hence, it is imperative to have a thorough comprehension of the complete pathophysiology of AD prior to directing attention toward the creation of new drugs. Furthermore, there is a shift in focus within AD drug development from treatment to prevention. Recent approaches appear to prioritize reducing the generation, aggregation, and misfolding of Aβ proteins and tau, while also increasing the removal of toxic aggregate or misfolded versions of these proteins. These tactics are based on earlier clinical and nonclinical research which warrants further investigation and exploration.

## Author contributions

AA: Conceptualization, Data curation, Formal analysis, Funding acquisition, Investigation, Resources, Validation, Writing – original draft, Writing – review & editing. RA: Formal analysis, Investigation, Methodology, Resources, Validation, Writing – original draft, Writing – review & editing. SS: Data curation, Formal analysis, Methodology, Project administration, Writing – original draft, Writing – review & editing. MS: Data curation, Formal analysis, Investigation, Methodology, Project administration, Writing – review & editing. KK: Data curation, Investigation, Methodology, Project administration, Visualization, Writing – review & editing. VL: Data curation, Formal analysis, Investigation, Project administration, Visualization, Writing – review & editing. AS: Formal analysis, Investigation, Methodology, Resources, Validation, Writing – review & editing.
